# Large granular lymphocyte leukemia serum and corresponding hematological parameters reveal unique cytokine and sphingolipid biomarkers and associations with STAT3 mutations

**DOI:** 10.1002/cam4.3246

**Published:** 2020-07-25

**Authors:** Kristine C. Olson, Katharine B. Moosic, Marieke K. Jones, Paige M. K. Larkin, Thomas L. Olson, Mariella F. Toro, Todd E. Fox, David J. Feith, Thomas P. Loughran

**Affiliations:** ^1^ University of Virginia Cancer Center Charlottesville VA USA; ^2^ Department of Medicine Division of Hematology/Oncology University of Virginia School of Medicine Charlottesville VA USA; ^3^ Department of Pathology University of Virginia School of Medicine Charlottesville VA USA; ^4^ Health Sciences Library University of Virginia School of Medicine Charlottesville VA USA; ^5^ Department of Pharmacology University of Virginia School of Medicine Charlottesville VA USA; ^6^ Present address: Department of Pathology and Laboratory Medicine University of California Los Angeles Los Angeles CA USA

**Keywords:** hexosylceramides, macrocytic anemia, neutropenia, sphingomyelins

## Abstract

Large granular lymphocyte (LGL) leukemia is a rare hematological disorder with expansion of the T‐cell or natural killer (NK) cell lineage. Signal transducer and activator of transcription 3 (STAT3) exhibits somatic activating mutations in 30%‐40% of LGL leukemia cases. Transcriptional targets of STAT3 include inflammatory cytokines, thus previous studies have measured cytokine levels of LGL leukemia patients compared to normal donors. Sphingolipid metabolism is a growing area of cancer research, with efforts focused on drug discovery. To date, no studies have examined serum sphingolipids in LGL leukemia patients, and only one study compared a subset of cytokines between the T‐LGL and NK‐LGL subtypes. Therefore, here, we included both LGL leukemia subtypes with the goals of (a) measuring serum sphingolipids for the first time, (b) measuring cytokines to find distinctions between the subtypes, and (c) establishing relationships with STAT3 mutations and clinical data. The serum analyses identified cytokines (EGF, IP‐10, G‐CSF) and sphingolipids (SMC22, SMC24, SMC20, LysoSM) significantly different in the LGL leukemia group compared to normal donors. In a mixed STAT3 mutation group, D661Y samples exhibited the highest mean corpuscular volume (MCV) values. We explored this further by expanding the cohort to include larger groups of single STAT3 mutations. Male D661Y STAT3 samples had lower Hgb and higher MCV compared to wild type (WT) or Y640F counterparts. This is the first report examining large groups of individual STAT3 mutations. Overall, our results revealed novel serum biomarkers and evidence that D661Y mutation may show different clinical manifestation compared to WT or Y640F STAT3.

## Graphical abstract

Analysis of LGL leukemia serum identified cytokines (EGF, IP‐10, G‐CSF) and sphingolipids (SMC22, SMC24, SMC20, LysoSM) that were significantly different compared to normal donors. Comparing three groups of STAT3 mutation status (WT, Y640F, D661Y) in LGL leukemia patients showed the D661Y mutation group had higher mean corpuscular volume and lower hemoglobin compared to the other groups.

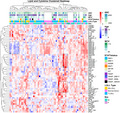

## 1 INTRODUCTION

Large granular lymphocyte (LGL) leukemia is a rare and chronic leukemia that typically exhibits expansion of CD3+CD8+CD57+ T‐cells or less frequently CD3−CD16+CD56+ natural killer (NK) cells.^1^ A rare aggressive subtype of NK‐LGL leukemia has a poor prognosis because patients do not respond to any treatments.^2^ Diagnosis of LGL leukemia is established by an increased expansion of lymphocytes by flow cytometry.^1^, ^2^ Clonality can be established for the T‐cell subtype by the T‐cell receptor (TCR) rearrangement test. NK cells do not express TCR, therefore, skewed killer‐cell immunoglobulin‐like receptors can suggest but not establish clonality.^1^, ^2^ Most cases of LGL leukemia are chronic and can be managed by a watch‐and‐wait approach.^2^ Symptoms such as anemia or neutropenia usually warrant treatment initiation of low‐dose immunosuppressants.^2^ Also, it is common for patients to have co‐occurrence of autoimmune disease, such as rheumatoid arthritis.^1^

It is well‐established that in LGL leukemia, the Janus kinase (JAK)––signal transducer and activator of transcription (STAT) pathway is hyperactivated, oftentimes by somatic *STAT* mutations.^3^, ^4^, ^5^, ^6^, ^7^, ^8^ Specifically, recurrent Signal transducer and activator of transcription 3 (STAT3) mutations in the Src‐homology 2 (SH2) domain have been reported in approximately 40% of both T‐ and NK‐LGL leukemia patients, with Y640F and D661Y the most commonly reported.^5^, ^7^, ^9^, ^10^, ^11^ STAT transcriptional target genes include inflammatory protein cytokines,^12^ and elevated cytokines are known to contribute to and worsen diseases.^13^ Furthermore, STAT3 mutations have been previously linked to elevated cytokines in LGL leukemia and the related disease, Felty's syndrome.^14^ Studies by our laboratory and others have measured various cytokines in LGL leukemia serum.^14^, ^15^, ^16^, ^17^, ^18^, ^19^, ^20^, ^21^, ^22^, ^23^, ^24^ Most of them focused on the more common subtype, T‐LGL leukemia, one examined NK‐LGL leukemia,^17^ and only one examined both subtypes.^24^ More studies are needed to determine whether there are distinguishing cytokines or other biomarkers between the two subtypes.

STAT3 mutation and elevated inflammatory cytokines are common to both LGL leukemia subtypes, therefore, other macromolecules may fundamentally distinguish the two subtypes. Previous work showed that sphingolipids play a role in survival of cytotoxic lymphocytes.^25^ Indeed, dysregulated sphingolipid metabolism is an active area of cancer research, including leukemia,^26^, ^27^ with great efforts to understand whether inhibitors of sphingolipid enzymes could be effective therapies.^28^, ^29^, ^30^, ^31^, ^32^ Hence, identifying different levels of sphingolipids in LGL leukemia or one of the subtypes could point to new and better prognostic tools and therapeutic targets.

Many LGL leukemia patients suffer from anemia or neutropenia. Several studies have reported that patients with a STAT3 mutation are more likely to have these cytopenias.^5^, ^7^, ^10^, ^11^, ^18^, ^33^, ^34^, ^35^ Yet, these studies have varying findings, most likely due to STAT3 mutation heterogeneity, environmental, or genetic influences. These studies usually report white blood cell (WBC) counts, hemoglobin (Hgb), and hematocrit (Hct). However, few studies have mentioned the incidence of macrocytic anemia^36^, ^37^ and no individual mean corpuscular volume (MCV) values have been reported in the literature. While known origins of macrocytic anemia, such as immunosuppressant treatment or a vitamin B12 deficiency^38^ may explain many cases, there are a subset of LGL leukemia patients who display elevated MCV without any known cause. Since STAT3 mutation status has been associated with cytopenias, we aimed to evaluate whether macrocytic anemia could be related to STAT3 mutation status.

We hypothesized that a serum cytokine and sphingolipid study that encompassed both T‐ and NK‐LGL leukemia compared to normal donors would provide a platform for biomarker discovery. In addition, it would be an opportunity to find novel correlations with clinical data based on STAT3 mutation status. Therefore, we measured 24 cytokines and 33 sphingolipids in the serum of 50 total LGL leukemia patients and 16 normal donors and assessed their clinical data and STAT3 mutation status. Overall, our results show that specific sphingolipids and cytokines are significantly different in LGL leukemia vs normal donors, and thus, may be relevant biomarkers. Additionally, grouping patients by specific STAT3 mutations and stratifying by sex is more informative than consolidating these groups to reveal differences in clinical manifestations.

## 2 MATERIALS AND METHODS

### 2.1 Human subjects and human serum samples

All human subjects were consented and samples were studied under IRB‐approved protocols for the LGL Leukemia Registry at the University of Virginia (IRB‐HSR#17000 “Large Granular Lymphocyte Leukemia Registry” and IRB #17070 “Pathogenesis of Large Granular Lymphocyte Leukemia”). The samples were isolated from confirmed LGL leukemia patients who showed an expanded LGL cell population with typical cell surface markers.^1^ Normal control sera were generously provided through a collaborative effort with Creative Testing Solutions, Tempe, Arizona.

### 2.2 Hematological parameters

Consented patients in the LGL Leukemia Registry provide test results corresponding to their research blood draw. Routine tests include a complete blood count (CBC). For this study, the following CBC values were utilized: WBC absolute count, total lymphocyte count (calculated by adding absolute lymphocyte count plus atypical lymphocyte count, if applicable), absolute neutrophil count (ANC), MCV, Hgb, and Hct. In a few cases the differential did not have an ANC value but instead included absolute granulocyte count, and therefore, the latter was used.

### 2.3 *STAT* mutation testing

The LGL Leukemia Registry conducts SH2 domain *STAT3* genotyping on patient samples if they have given genetic consent. Mutation analysis of the STAT3 SH2 domain was established since this is the hotspot that contains the majority of reported somatic mutations, and analysis of other parts of the gene sequence was not completed. *STAT* mutation testing was performed as previously described^4^ for the majority of the samples. Briefly, DNA from PBMCs was extracted (Anaprep system; BioChain), amplified with premade primers, and submitted for Sanger sequencing (Eurofins). For some samples, STAT3 mutation status had already been determined by whole genome sequencing, or a CLIA‐approved mutation profiling test, or a droplet digital PCR assay.^39^

### 2.4 Cohorts in this study

**FIGURE 1:**
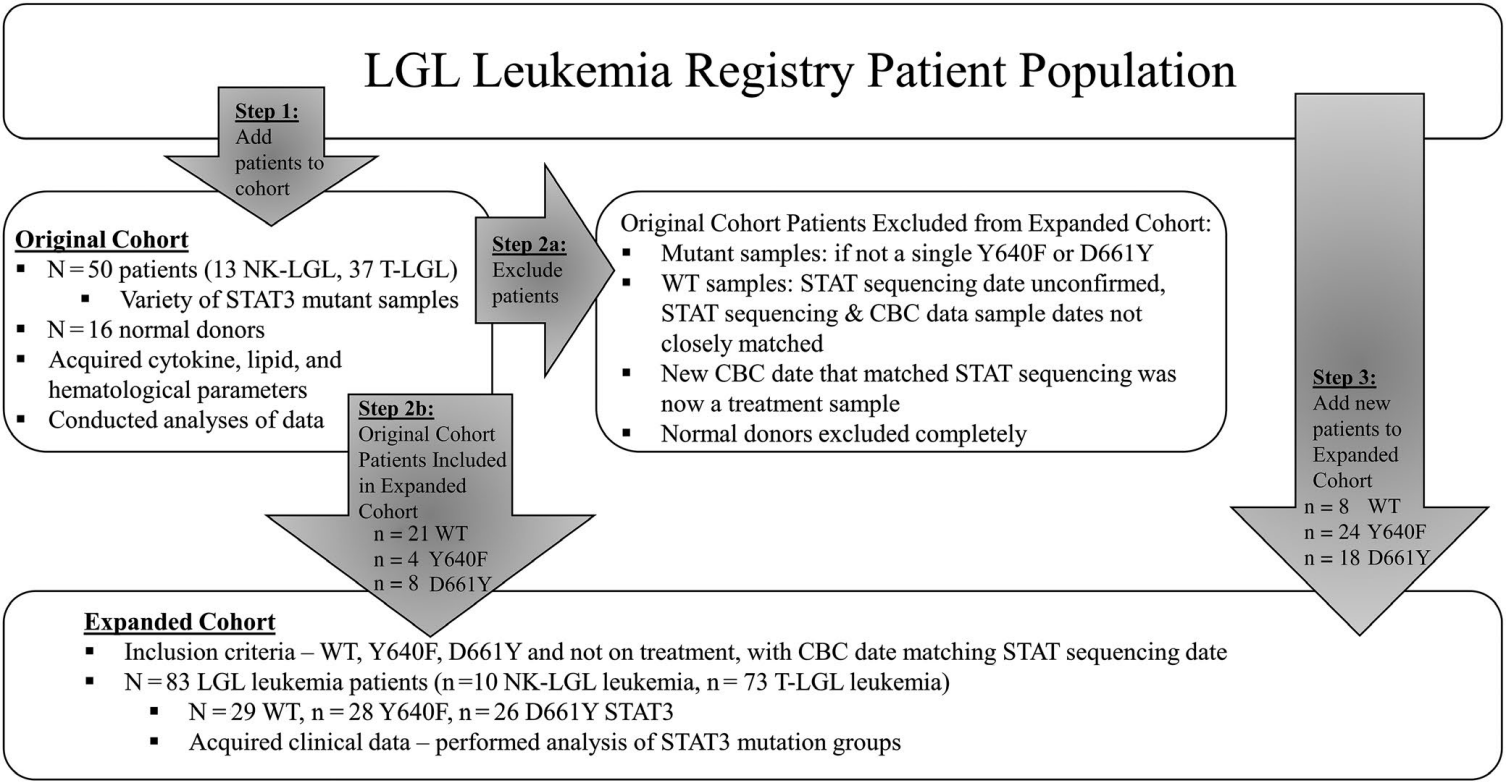
Flow chart showing selection of Original Cohort and Expanded Cohort used in this study. The selection process and data acquired for each cohort are displayed as a flow chart. Additional details can be found in Section 2

Refer to Figure 1 for a synopsis of cohorts. The patient serum samples selected for this study are referred to as the *Original Cohort*. The Original Cohort also had normal donor samples for demographic and research measurements, but not clinical measurements. All samples used were taken from a “no treatment” date. If a patient previously had treatment, a standard washout period had passed to warrant its classification as “no treatment.” Treatment refers to immunosuppressant treatment such as methotrexate, cyclophosphamide, cyclosporine, high doses of prednisone, or other immunosuppressants prescribed for autoimmune disease. Only immunosuppressive treatments were considered in the inclusion process. Corresponding clinical data (blood counts) were acquired for the date matching the serum sample, except in one case, the research sample and CBC data were acquired from two separate blood samples collected approximately 6 weeks apart. After analyses showed a potential relationship between the D661Y STAT3 mutation and elevated MCV, an *Expanded Cohort* was used to rigorously confirm this finding. The goal of the Expanded Cohort was to compare three groups: STAT3 wild type (WT), Y640F, or D661Y patients. Therefore, patients from the Original Cohort with mutations other than Y640F or D661Y were excluded, but a subset who met criteria (n = 21 WT, n = 4 Y640F, n = 8 D661Y) were included in the Expanded Cohort. Then, additional patients were added to the three aforementioned groups to establish the Expanded Cohort. Selection of patients not on treatment at the time of *STAT* testing provided a total of 28 Y640F and 26 D661Y patients in the Expanded Cohort. WT patients were selected by working backward in the Registry, from the most recently genotyped, and excluding patients on treatment, leading to 29 total WT patients. It has been documented that an LGL leukemia patient's STAT3 mutation status can change over time.^39^ Therefore, it was important to gather data on STAT mutational status and CBC results for the same sample date, and not greater than 1 month difference in rare cases that lacked an exact match. Two exceptions were a 6 month difference and a “never‐treated” patient with WT STAT3 status who was tested for mutations 14 months before the clinical data were recorded. Many patients from the Original Cohort had exact date matches for serum sample, CBC results, and STAT3 testing. However, those included in the Expanded Cohort had their CBC results updated to match STAT3 testing date, if necessary. Follow‐up analysis on a subset of WT (n = 11), Y640F (n = 11), and D661Y (n = 12) used MCV and Hgb values from the most recent CBC to compare to the previous values.

### 2.5 Cytokine analysis

Banked LGL leukemia patient or normal donor serum samples were retrieved from −80°C storage and analyzed by the UVA Flow Cytometry Core using a Luminex MAGPIX bead‐based multiplex analyzer. The serum samples were analyzed on six panels (Milliplex Human Multiplex Assay) for a total of 25 cytokines assayed: *Panel 1* 14‐plex (interleukin [IL]‐8, interferon [IFN]‐γ, IFN‐α2, IL‐6, TNF‐related apoptosis‐inducing ligand [TRAIL], macrophage inflammatory protein [MIP]‐1β, IL‐1 receptor α [IL‐1Rα], MIP‐3β, epidermal growth factor [EGF], FMS‐like tyrosine kinase 3 ligand [Flt‐3L], granulocyte‐colony stimulating factor [G‐CSF], IL‐10, stromal cell‐derived factor 1 alpha and beta [SDF‐1α+β], IL‐18), *Panel 2* 1‐plex (regulated on activation normal T expressed and secreted [RANTES]), *Panel 3* 3‐plex (transforming growth factor [TGF]‐β1, TGFβ2, TGF‐β3), *Panel 4* 4‐plex (soluble vascular cell adhesion protein 1 [sVCAM‐1], soluble intercellular cell adhesion protein 1 [sICAM], soluble Fas [sFas], soluble Fas ligand [sFasL]), *Panel 5* 1‐plex (eosinophil chemotactic protein 2 [Eotaxin‐2]), *Panel 6* 2‐plex (interferon gamma‐induced protein 10 [IP‐10], monokine induced by gamma interferon [MIG]). For any cytokine values outside of the quantitative range, the minimum or maximum range values were utilized. The results for TGF‐β3 were below the limit of detection for all serum samples, therefore, these data were omitted from the manuscript.

### 2.6 Sphingolipid analysis

Lipids were extracted from 20 µL of serum using an azeotrophic mix of isopropanol:water:ethyl acetate (3:1:6; v:v:v). Internal standards (10 pmol of d17 long‐chain bases and C12 acylated sphingolipids) were added to samples at the onset of the extraction procedure. Extracts were separated on a Waters I‐class Acquity UPLC chromatography system. Mobile phases were (A) 60:40 water:acetonitrile and (B) 90:10 isopropanol:methanol with both mobile phases containing 5 mmol/L of ammonium formate and 0.1% of formic acid. A Waters C18 CSH 2.1 mm ID × 10 cm column maintained at 65°C was used for the separation of the sphingoid bases, 1‐phosphates, and acylated sphingolipids. The eluate was analyzed with an inline Waters TQ‐S mass spectrometer using multiple reaction monitoring. All data reported are based on monoisotopic mass and are represented as pmol/mL serum.

### 2.7 Heatmap analysis

A heatmap of the lipid and cytokine data collected for the LGL leukemia samples and normal donors was generated using the pheatmap package in R.^40^ The serum concentration values were log 10 transformed, centered, and scaled, and data were clustered by both row and column using Euclidean distance using complete linkage clustering. Clinical data, STAT3 mutation status, LGL leukemia type, and sex were not incorporated into the clustering algorithm, but were included when annotating the samples. In the annotation labels, ANC, Hgb, and MCV are represented as continuous variables, whereas the remainder of the labels are discrete. The code created for generating the heatmap can be accessed at: https://github.com/kbm4dd/Cancer‐Medicine‐Manuscript‐2020

### 2.8 Statistical analysis

Concentrations of log‐transformed serum cytokines and sphingolipids were each analyzed between normal donors, NK‐LGL, and T‐LGL leukemia samples using one‐way ANOVA and between NK‐LGL vs T‐LGL leukemia samples using an unpaired Student's *t* test in R version 3.6.1. *P*‐values were corrected for multiple testing across all biomarkers (n = 57) via the Holm‐Sidak method. Analysis of the Original Cohort blood counts, separated into WT vs mutant STAT3 groups utilized unpaired Student's *t* tests. *P*‐values for these tests were corrected for six tests using the Holm‐Sidak method. The Original Cohort data for MCV, Hgb, and Hct were further divided by sex and analyzed via two‐way ANOVAs corrected for multiple testing over three tests (correction for interaction, sex, and genotype global *P*‐values). The post hoc Sidak *P*‐values are also reported. Regression models were built to explain log transformed serum cytokines or sphingolipid levels by MCV and an interaction with STAT3 mutant status (R version 3.6.1). Analyses of the Expanded Cohort blood count parameters were conducted as one‐way ANOVAs, with the white cell and red cell parameter global *P*‐values each corrected over three tests using the Holm‐Sidak method. The Expanded Cohort data for MCV, Hgb, and Hct were further divided by sex and analyzed via two‐way ANOVAs corrected for multiple testing over three tests as above. The post hoc Sidak *P*‐values are also reported. MCV vs Hgb values were plotted for WT, Y640F, and D661Y in the Expanded Cohort and a chi‐square test was performed to analyze the proportion of values in or out of the established normal ranges. Follow‐up of the Expanded Cohort used paired Student's *t* tests to compare between initial and follow‐up samples within each STAT3 mutation group. Analyses were conducted in GraphPad Prism software version 8 unless otherwise stated.

## 3 RESULTS

### 3.1 Demographics and clinical information in the Original Cohort of LGL leukemia patients

The LGL Leukemia Registry provides an opportunity to merge research data acquired from patient samples with their corresponding clinical data. The Original Cohort and normal donor samples used for the first part of this study have demographic and selected clinical data listed in Table 1. For n = 12 NK‐LGL leukemia and n = 33 T‐LGL leukemia patients, clinical data were available on the serum sample date. We determined the mean, standard deviation (SD), and range for WBC count, total lymphocytes, ANC, Hgb, and Hct from these patients. Twenty‐one patients had a STAT3 SH2 domain mutation, with the largest groups representing the common mutations of D661Y (n = 8) and Y640F (n = 4). This cohort of LGL leukemia patients with both subtypes of LGL leukemia was utilized for comparison to normal donors.

**TABLE 1:** Composite table of LGL leukemia and normal donor Original Cohort demographic and clinical information. All LGL leukemia patients chosen for this study had a confirmed diagnosis and were not on immunosuppressive treatment at the time of sample collection. The table lists data according to three groups: normal donors, NK‐LGL leukemia, and T‐LGL leukemia. STAT3 mutation status is listed for the LGL leukemia patients, with specific mutation breakdowns. Blood count data were available for n = 12 NK‐LGL leukemia and n = 33 T‐LGL leukemia patients. Blood count data were measured from the same research sample draw date, except one case where these were 6 wk apart. Five additional patients are missing blood count data due to these dates being too far apart from the research sample draw. The normal donors did not have blood count data, therefore, the normal reference ranges according to the University of Virginia clinical laboratory standards are listed. All parameters are reported as mean ± SD, with the range listed in parentheses

Sample group	Normal donors	NK‐LGL leukemia	T‐LGL leukemia
n	16	13	37
Mean age ± SD (Range)	59.1 ± 12.9 (45‐79)	62.5 ± 13.7 (32‐81)	60.6 ± 14.6 (22‐88)
Sex	Female, n = 8	Female, n = 5	Female, n = 19
	Male, n = 8	Male, n = 8	Male, n = 18
STAT3 mutation status	Undetermined	**WT, n = 8** **Mutated, n = 5** n = 1 S614R N647I K658R D661I D661Y	**WT, n = 21** **Mutated, n = 16** n = 1 Y640F/Q643H Y640F/I659L Y640F/D661Y n = 2 N647I n = 4 Y640F n = 7 D661Y
WBC	Reference range 4.00‐11.00 k/µL	n = 12 8.96 ± 4.98 (2.24‐21.00)	n = 33 9.63 ± 9.60 (1.05‐47.50)
Total lymphocytes	Reference range 1.00‐5.00 k/µL	n = 12 6.12 ± 5.03 (0.67‐19.10)	n = 33 7.18 ± 8.87 (0.38‐43.70)
ANC	Reference range 1800‐8000 cells/µL	n = 12 2107 ± 909 (800‐4100)	n = 33 1780 ± 1252 (180‐4940)
Hgb	Reference range	Overall, n = 12 12.7 ± 1.3 (10.8‐15.2)	Overall, n = 33 12.2 ± 2.3 (5.7‐15.9)
	12‐16 g/dL (F)	Female, n = 5 12.4 ± 1.1 (11.0‐14.1)	Female, n = 17 11.8 ± 2.1 (5.7‐14.1)
	14‐18 g/dL (M)	Male, n = 7 12.8 ± 1.4 (10.8‐15.2)	Male, n = 16 12.7 ± 2.5 (8.3‐15.9)
Hct	Reference range	Overall, n = 12 37.7 ± 3.8 (32.4‐45.2)	Overall, n = 33 36.3 ± 6.5 (16.6‐47.4)
	35.0%‐47.0% (F)	Female, n = 5 36.7 ± 2.6 (34.1‐41.0)	Female, n = 17 35.2 ± 6.2 (16.6‐43.0)
	40.0%‐52.0% (M)	Male, n = 7 38.4 ± 4.5 (32.4‐45.2)	Male, n = 16 37.5 ± 6.9 (25.8‐47.4)

Abbreviations: ANC, absolute neutrophil count; Hct, hematocrit; Hgb, hemoglobin; LGL, large granular lymphocyte; NK, natural killer; WBC, white blood cell; WT, wild type.

### 3.2 Novel serum cytokines and sphingolipids distinguish LGL leukemia patients from normal donors

**FIGURE 2:**
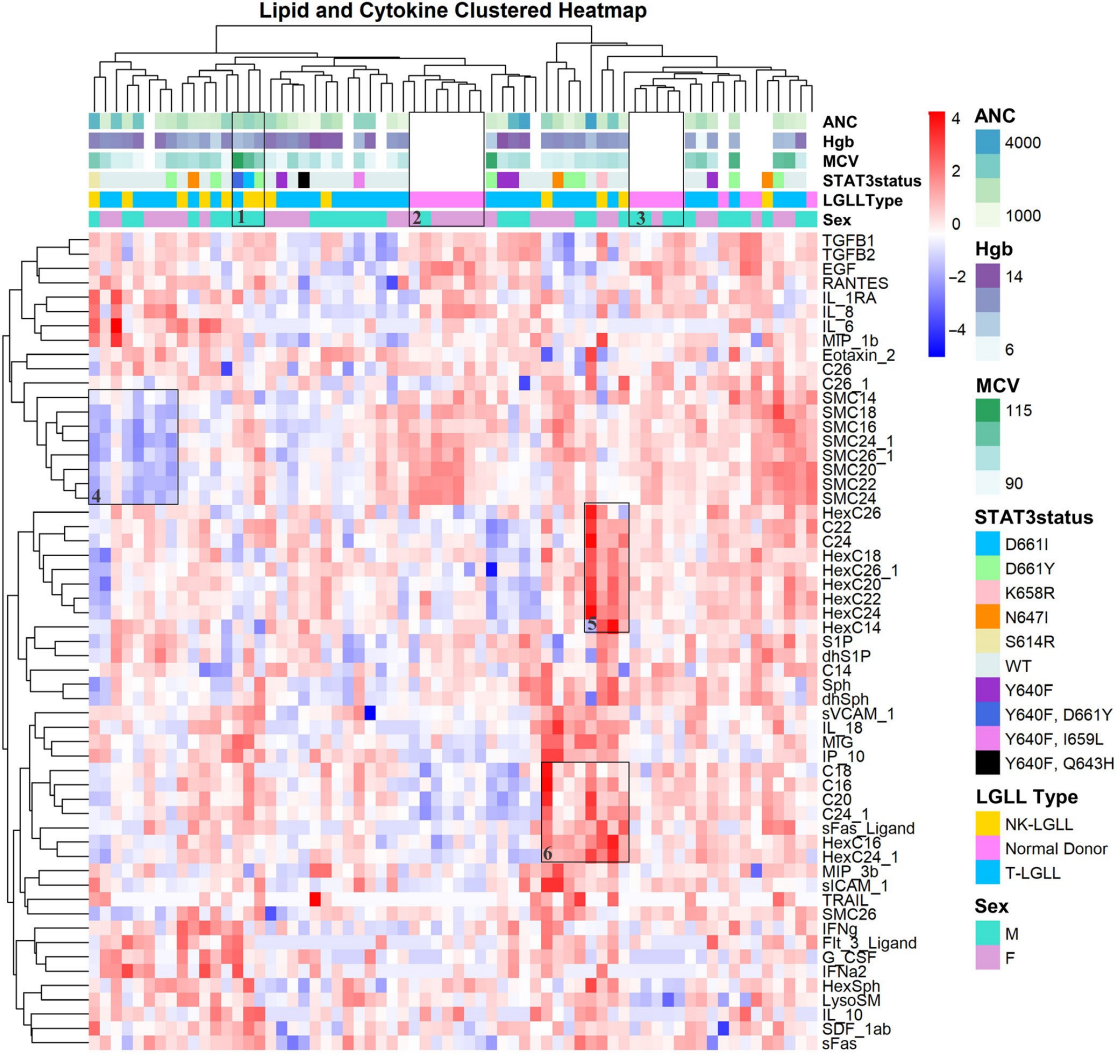
Heatmap of large granular lymphocyte (LGL) leukemia and normal donor serum samples with cytokine and sphingolipid values. A heatmap was generated for LGL leukemia samples and normal donors and grouped using hierarchical clustering. Serum cytokine and sphingolipid values were log 10 transformed and scaled prior to this analysis. Red and blue indicate high and low levels, respectively, of cytokine or sphingolipid relative to the mean value in each row. Additional information such as clinical data, STAT3 mutation status, LGL leukemia type, and sex are indicated on the top of the heatmap and were not a part of the clustering algorithm. These parameters are defined in the legend on the right and explored in more detail in later figures and tables. The clinical data for the patients matched exactly with the serum sample date, except for one case where it was different by 6 weeks. Five patients do not have clinical data listed since their blood count data were too far off from the serum sample date. These samples are shown as white. The normal donor samples do not have clinical or STAT3 data, therefore, these areas are also white. Hierarchal clustering analysis based on Euclidean distance demonstrates clustering and is annotated by numbered boxes (discussed in Section 3)

In the Original Cohort, we measured 24 cytokines and 33 sphingolipids in LGL leukemia (n = 13 NK, n = 37 T) and normal donor serum (n = 16), with the goal of finding biomarkers that distinguish between the subtypes. The mean, SD, and range for each of these parameters can be found in Tables S1 and S2 (cytokines and sphingolipids, respectively). Next, a heatmap (Figure 2) was generated to visualize the LGL leukemia patients and normal donors in columns labeled with ANC, Hgb, MCV, STAT3 status, LGL leukemia subtype, and sex. The rows show relative levels of cytokines and sphingolipids. Each parameter was mean centered and scaled. Hierarchical clustering was utilized to visualize how LGL leukemia and normal donors as well as sphingolipid and cytokine biomarkers grouped together. Columns cluster similar samples, whereas rows group the most similar biomarkers. Of interest, the normal donors largely clustered together in two groups (boxes 2 and 3) with one predominantly female and the other predominantly male. The heatmap showed a distinct cluster of patients with low sphingomyelins (box 4), while the normal donors and most other LGL leukemia patients showed higher sphingomyelin levels. A cluster of patients exhibited elevated ceramides and sFas ligand (box 6), and some of these patients also had uniform elevation of hexosylceramides (box 5). Finally, a small group of male patients with a mutation at the D661 position of STAT3 had elevated MCV (box 1). Therefore, cluster analysis allowed us to identify groups of patients exhibiting similar trends, driving further analysis of these potential biomarkers and subgroups of patients.

**FIGURE 3:**
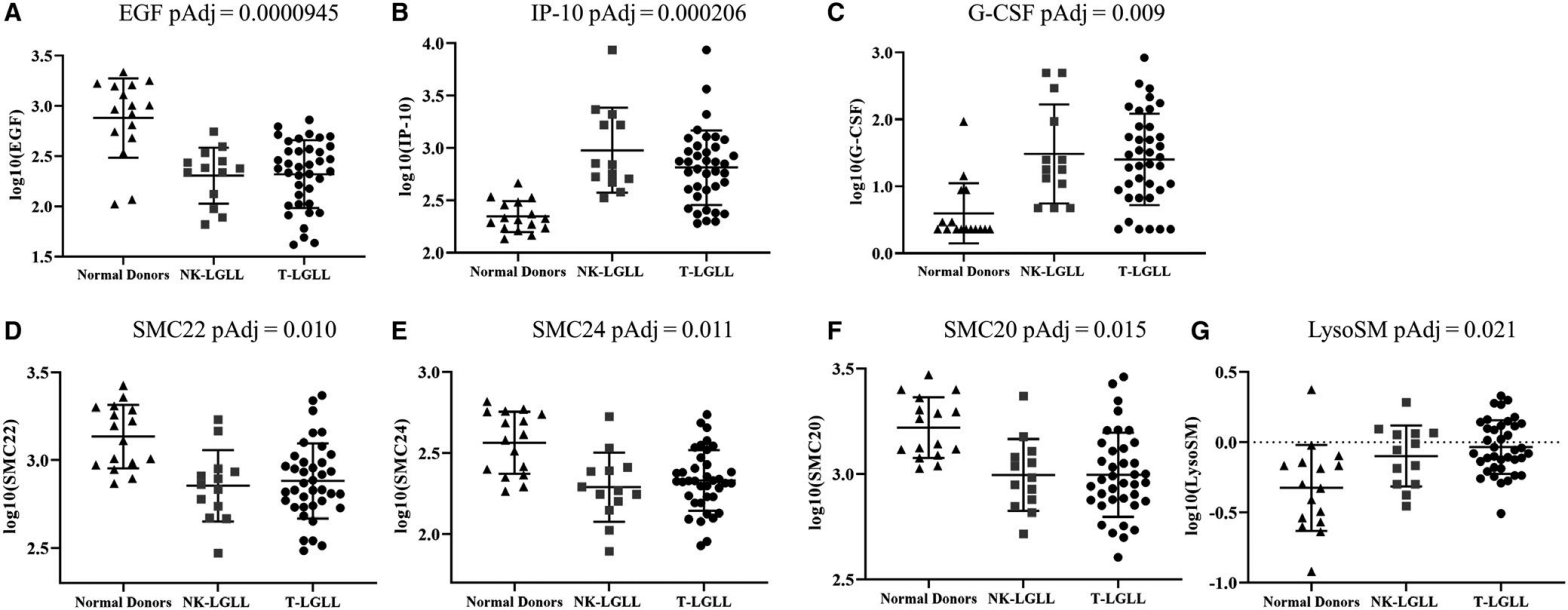
Summary of the seven significant serum biomarkers in large granular lymphocyte (LGL) leukemia compared to normal donor samples. Strip plots with summary (mean ± SD) show cytokines (EGF (A), IP‐10 (B), and G‐CSF (C)) or sphingolipids (SMC22 (D), SMC24 (E), SMC20 (F), and LysoSM (G)) that were significantly different between LGL leukemia (n = 13 NK, n = 37 T) serum and normal donor (n = 16) serum. Adjusted *P*‐values (*P*Adj; after multiple testing correction) are reported (values for all biomarkers are in Table S3)

Next, we completed individual analysis of each parameter, using ANOVA and multiple testing correction. This strategy pinpointed seven cytokine and sphingolipid parameters (EGF, IP‐10, G‐CSF, SMC22, SMC24, SMC20, and LysoSM) that were significantly different between LGL leukemia subtypes and normal donor samples (Table S3). Cytokines that were previously reported to be elevated in LGL leukemia, including sFas ligand and IL‐18^16^, ^21^, ^22^ were also significant in our cohort before multiple testing correction. Figure 3 shows each of these seven parameters, with LGL leukemia patients divided by subtype. EGF (Figure 3A), IP‐10 (Figure 3B), and G‐CSF (Figure 3C) were the top significant differences between LGL leukemia and normal donors. EGF was decreased while IP‐10 and G‐CSF were increased in LGL leukemia samples compared to normal donors. These cytokines are novel, as only one study previously reported these three cytokines as significantly different in a smaller cohort of NK‐LGL leukemia,^17^ and to the best of our knowledge, this has never been reported in T‐LGL leukemia. The sphingolipids SMC22 (Figure 3D), SMC24 (Figure 3E), SMC20 (Figure 3F), and LysoSM (Figure 3G) were significantly different in LGL leukemia compared to normal donors. SMC22, SMC24, and SMC20 were all decreased while LysoSM was increased in LGL leukemia samples compared to normal donors. Of interest, SMC22, SMC24, and SMC20 also clustered together in the heatmap (Figure 2). This is the first report of sphingolipid measurements in LGL leukemia patient serum. No parameters held up as significantly different between NK vs T‐LGL leukemia subtypes after multiple testing correction. NK‐LGL leukemia sera exhibited ceramide C22 levels that were elevated relative to normal donors and T‐LGL leukemia although just short of statistical significance (*P* = .060; Figure S1; Table S4). Taken together, we report that the cytokines EGF, IP‐10, and G‐CSF and the sphingolipids SMC22, SMC24, SMC20, and LysoSM are significantly different in LGL leukemia compared to normal donor serum. These are all noncanonical biomarkers of LGL leukemia and, due to our rigorous statistical testing, we propose that they represent biologically relevant directions for new research.

### 3.3 MCV from the Original Cohort shows differences when compared by STAT3 mutation status

**FIGURE 4:**
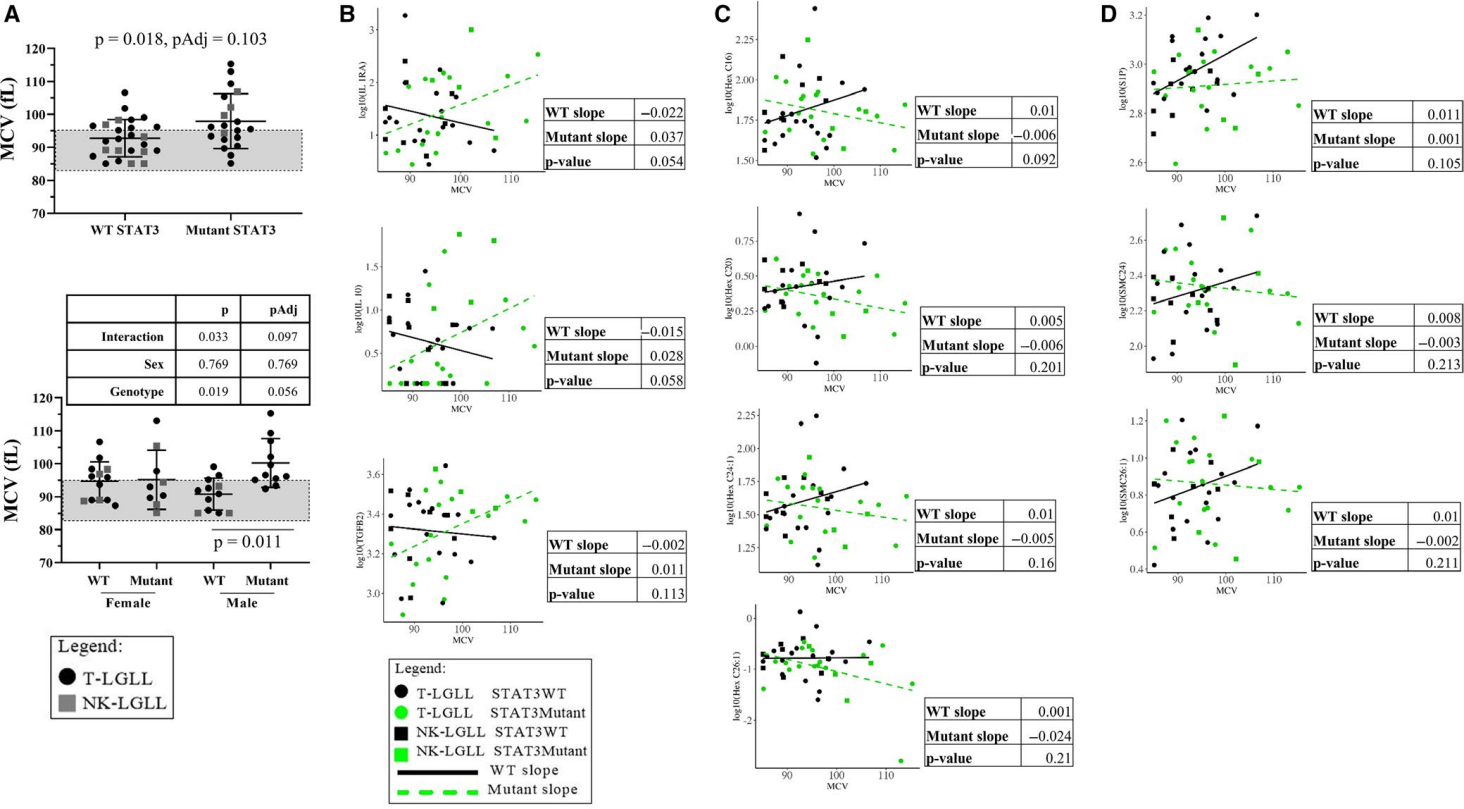
Serum mean corpuscular volume (MCV) values and regression analysis of cytokine or sphingolipid measurements in wild type (WT) or mutant STAT3 patient groups. Strip plots with summary (mean ± SD) show MCV data from the Original Cohort according to WT or mutant STAT3 status. The normal reference range for MCV is indicated by the gray box (MCV = 83.0‐95.0 fL). An MCV value of >95 fL is above normal reference range and indicates macrocytic anemia. A (upper graph), A *t* test showed MCV was significantly higher in STAT3 mutant compared to WT STAT3 patient samples, but not significant after multiple testing correction (Holm‐Sidak); six of the samples above the mean were D661Y mutants; (lower graph) MCV data were stratified by sex. Two‐way ANOVA results for genotype main effect, sex main effect, and interaction are shown in the table above the graph. Significant multiple comparisons (Sidak method) are shown within the graph. B‐D, Regression to determine relationship between cytokine and sphingolipid levels and MCV with an interaction term for STAT3 mutation status was plotted for the top 10 parameters with different slopes between STAT3 WT vs STAT3 mutant groups. Slopes and *P*‐values for WT and mutant STAT3 are reported next to each graph. No *P*‐values were significant for any of these analyses, therefore, no multiple testing corrections were applied. Cytokines and sphingolipids measured from serum samples matched the blood draw date of the MCV data. Refer to the legends for information on color and shape coding

Next, we analyzed whether hematological parameters (Table 1) varied according to STAT3 mutation status. MCV is a hematological parameter with limited studies in LGL leukemia. We observed an interesting pattern in MCV (Figure 4A, top graph), where mutant STAT3 samples tended to have an elevated MCV, and in particular this observation was driven by the male samples (Figure 4A, bottom graph). Twelve patients in the mutant group (roughly 60%) had an MCV value above the normal range. Of these 12, six of the patients had a D661Y STAT3 single mutation (50%). The other six mutations represented were: n = 1 of Y640F; D661I; S614R; N647I and n = 1 of double mutant Y640F/I659L and Y640F/D661Y. Of interest, the patients with the five highest data points (range of 115.3‐105.4 fL), all exhibited mutation at the D661 residue (in descending order: Y640F/ D661Y; D661Y, D661Y, D661I, and D661Y) (Figure 4A). Other hematological parameters were not significantly different between the groups (Figure S2A‐G). Overall, these data suggest that MCV, a parameter of red blood cell size not previously well‐studied in LGL leukemia, correlates with D661 mutations in STAT3.

### 3.4 Regression analysis of MCV and cytokine or sphingolipid measurements according to STAT3 mutation status

We then used a regression to determine what relationships exist between serum biomarkers, MCV, and STAT3 mutation status. While these analyses did not yield any relationships that significantly differed by STAT3 status (Table S5), we report the 10 cytokines and sphingolipids that exhibit the most different slopes between WT and mutant STAT3 samples. This list includes three cytokines (IL‐1RA, IL‐10, and TGFβ2, Figure 4B), four hexosylceramides (HexC16, HexC20, HexC24:1, and Hex C26:1, Figure 4C), and three additional sphingolipids (S1P, SMC24, and SMC26:1, Figure 4D). Furthermore, we report the bottom 10 with least significant interactions (Figure S3A‐C), representing biomarkers that trend the same way in LGL leukemia irrespective of STAT3 mutation status. Taken together, these data provide potential downstream molecular targets of STAT3 mutations in LGL leukemia to be explored in future laboratory studies as well as larger patient cohorts.

### 3.5 An Expanded Cohort demonstrates D661Y STAT3 mutation is more common in macrocytic anemia

The observation that D661Y STAT3 is associated with increased MCV (Figure 4A) led us to expand our patient cohort to substantiate our findings. Y640F and D661Y are somatic activating mutations that represent the most frequently occurring mutations in both Registry and literature reports.^5^, ^7^, ^18^, ^33^ Therefore, the goal of the Expanded Cohort was to compare three STAT3 groups: WT, Y640F, or D661Y patients. We kept WT (n = 21), Y640F (n = 4), and D661Y (n = 8) patients from the Original Cohort (other STAT3 mutations were excluded), then added additional patients to the three groups (see Figure 1 and Section 2 for more details). The Expanded Cohort encompassed n = 29 WT, n = 28 Y640F, and n = 26 D661Y patients to enable a rigorous comparison of hematological parameters between defined mutational groups. The demographic and clinical data are reported in Table 2.

**TABLE 2:** Composite table of LGL leukemia Expanded Cohort demographic and clinical information. LGL leukemia patients from the Original Cohort who had the STAT3 status of WT, Y640F, and D661Y were kept and additional patients were added to each of these groups to create the Expanded Cohort. The additional patients had a confirmed diagnosis and were not on immunosuppressive treatment on the date the clinical measurements were acquired. The table lists the patients as three groups: WT, Y640F, and D661Y STAT3. Blood count data were available for all patients and are listed along with reference ranges and units for each parameter. The clinical data matched the date of STAT3 mutation testing, with few exceptions (see Section 2). Patients from the Original Cohort whose STAT3 mutation testing was done on a date other than that of their serum sample had their information updated to match the STAT3 date. All parameters are reported as mean ± SD, with the range listed in parentheses

Expanded cohort	WT STAT3	Y640F STAT3	D661Y STAT3
n	29	28	26
Mean age ± SD (Range)	59.0 ± 12.9 (32‐88)	51.0 ± 15.5 (22‐74)	61.7 ± 14.3 (25‐88)
Sex	Female, n = 13	Female, n = 16	Female, n = 12
	Male, n = 16	Male, n = 12	Male, n = 14
LGL leukemia subtype	T‐LGLL, n = 22	T‐LGLL, n = 28	T‐LGLL, n = 23
	NK‐LGLL, n = 7		NK‐LGLL, n = 3
**WBC**			
Reference range 4.00‐11.00 k/µL	8.07 ± 5.26 (1.05‐23.05)	5.20 ± 1.80 (2.67‐11.06)	10.5 ± 13.9 (1.60‐63.46)
**Total lymphocytes**			
Reference range 1.00‐5.00 k/µL	5.00 ± 4.63 (0.38‐19.13)	3.42 ± 1.70 (0.71‐6.97)	8.60 ± 13.45 (0.62‐60.29)
**ANC**			
Reference range 1800‐8000 cells/µL	2399 ± 1753 (180‐7300)	1241 ± 1011 (70‐4040)	1162 ± 702 (10‐2500)
**Hgb**			
Reference range	Overall 13.1 ± 1.8 (5.7‐15.9)	Overall 12.9 ± 2.2 (8.0‐16.7)	Overall 11.7 ± 2.1 (8.1‐15.9)
12‐16 g/dL (female)	Females 12.1 ± 2.1 (5.7‐14.1)	Females 12.4 ± 1.8 (8.3‐14.8)	Females 11.7 ± 1.3 (9.4‐14.0)
14‐18 g/dL (male)	Males 13.9 ± 1.2 (11.6‐15.9)	Males 13.5 ± 2.6 (8.0‐16.7)	Males 11.8 ± 2.7 (8.1‐15.9)
**Hct**			
Reference range	Overall 38.8 ± 5.4 (16.6‐45.9)	Overall 38.2 ± 6.5 (24.1‐47.4)	Overall 35.0 ± 6.1 (24.3‐47.7)
35.0%‐47.0% (female)	Females 36.5 ± 6.5 (16.6‐43.0)	Females 36.8 ± 5.7 (24.1‐44.0)	Females 35.5 ± 4.2 (27.6‐43.7)
40.0%‐52.0% (male)	Males 40.8 ± 3.4 (32.8‐45.9)	Males 40.0 ± 7.4 (24.5‐47.4)	Males 34.6 ± 7.5 (24.3‐47.7)

Abbreviations: ANC, absolute neutrophil count; Hct, hematocrit; Hgb, hemoglobin; LGL, large granular lymphocyte; WBC, white blood cell; WT, wild type.

**FIGURE 5:**
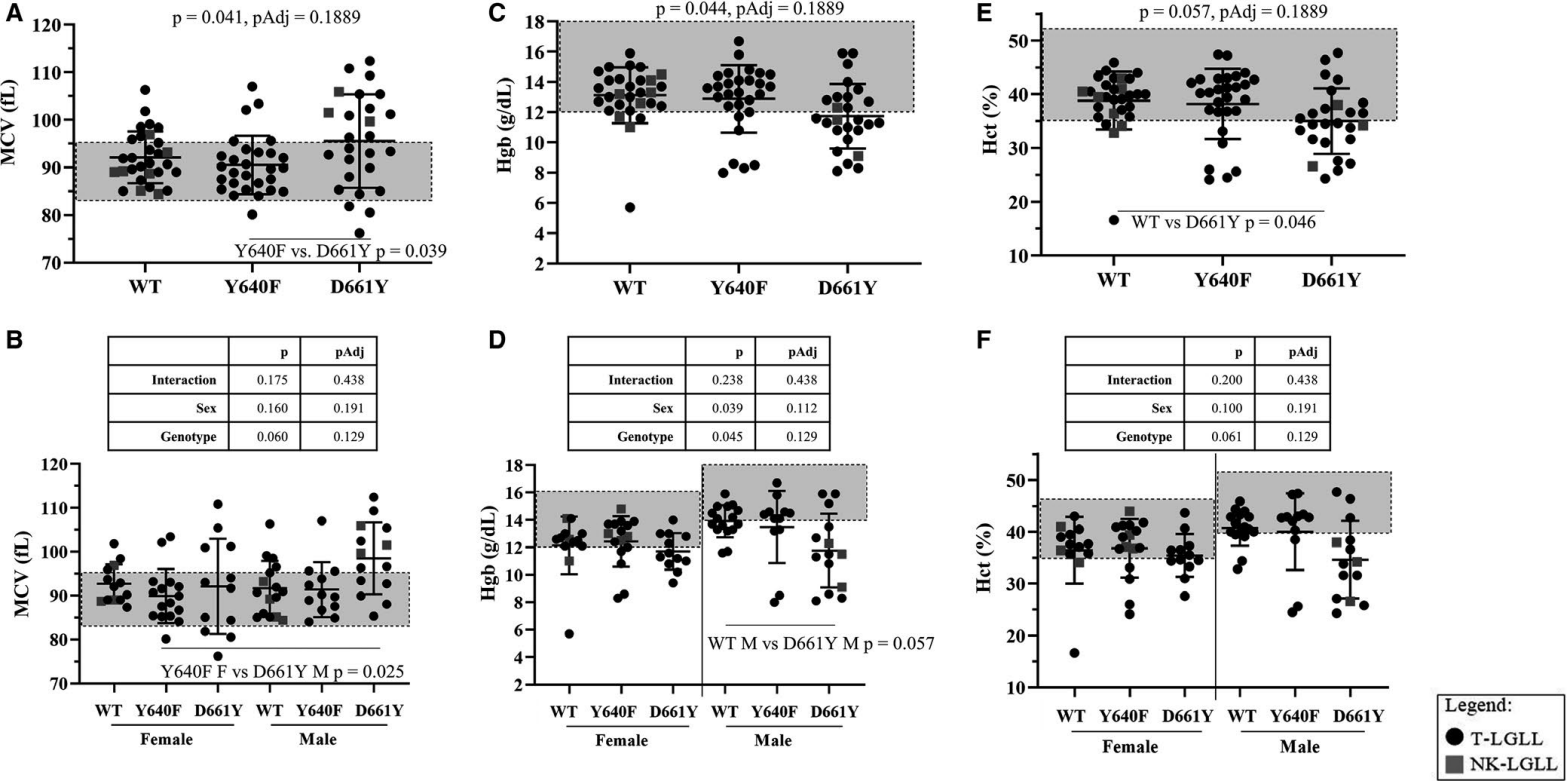
Mean corpuscular volume (MCV), hemoglobin (Hgb), and hematocrit (Hct) data from the Expanded Cohort according to STAT3 mutation status. Strip plots with summary (mean ± SD) show MCV, Hgb, and Hct according to STAT3 mutation status. Normal reference ranges are indicated by the gray boxes (MCV = 83.0‐95.0 fL, Hgb = 12‐16 g/dL for females and 14‐18 g/dL for males, Hct = 35.0%‐47.0% for females and 40.0%‐52.0% for males). A Hgb value of <12 g/dL or <14 g/dL is considered below normal reference range and indicates anemia in females or males, respectively. One‐way ANOVAs comparing STAT3 mutation groups are reported as P and PAdj (Holm Sidak) at the top of the graphs for MCV (A), Hgb (C), and Hct (E). Two‐way ANOVA results for genotype main effect, sex main effect, and interaction are shown in the table above the graphs for MCV (B), Hgb (D), and Hct (F). Significant multiple comparisons (Sidak method) are shown within the graphs (panels A, B, D, E). Refer to the legend for information on color and shape coding

Red blood cell‐related CBC data relative to STAT3 mutation status are reported in Figure 5. When analyzed by mutation status, MCV (Figure 5A) and Hgb (Figure 5C) had significant global *P*‐values and Hct (Figure 5E) was almost significant (*P* = .057). Rigorous multiple testing correction (Holm‐Sidak) resulted in nonsignificant *P*‐values for each of these comparisons. MCV (Figure 5A) showed a significant difference between the Y640F and D661Y mutant groups (*P* = .039, one‐way ANOVA, Sidak multiple comparisons). We found that when stratifying MCV by sex, data points were most variable in both the D661Y female and male groups compared to Y640F or WT STAT3 groups (Figure 5B). The D661Y group had 15/26 (58%) Hgb data points below 12 g/dL (Figure 5C). When split by sex, we observed the male D661Y samples were driving this finding, with a near significant difference between the WT and D661Y male groups (Figure 5D, *P* = .057; two‐way ANOVA, Sidak multiple comparisons). Strikingly, 11 of the 14 male D661Y samples (79%) were below the Hgb bottom reference range value of 14 g/dL (Figure 5D). Hct was significantly decreased (*P* = .046) in D661Y compared to WT (Figure 5E), and stratifying by sex showed this finding was driven by male D661Y samples (Figure 5F). Taken together, male D661Y STAT3 mutant samples were more likely to have high MCV and low Hgb and Hct compared to their male counterparts in the WT or Y640F STAT3 groups. No such differences were observed in the female groups.

**FIGURE 6:**
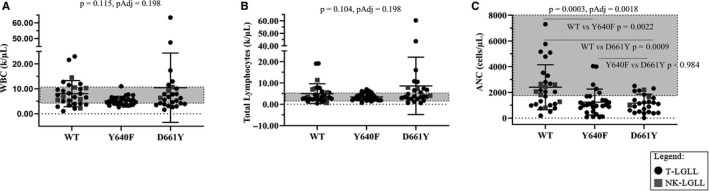
White blood cell (WBC), total lymphocytes, and absolute neutrophil count (ANC) from the Expanded Cohort according to STAT3 mutation status. Strip plots with summary (mean ± SD) show WBC, total lymphocytes, and ANC according to STAT3 mutation status. Normal reference ranges are indicated by the gray boxes (WBC = 4.00‐11.00 k/µL, total lymphocytes = 1.00‐5.00 k/µL, and ANC = 1800‐8000 cells/µL). One‐way ANOVAs comparing the STAT3 mutation groups are reported as *P* and *P*Adj (Holm‐Sidak) at the top of the graphs for WBC (A), total lymphocytes (B), and ANC (C). Significant multiple comparison (Sidak method) are shown in panel C. Refer to the legend for information on color and shape coding

Next, we examined additional hematological parameters (Figure 6), for association with STAT3 mutation status. No significant differences were observed for WBC and total lymphocyte count among the WT, Y640F, and D661Y groups (Figure 6A,B, respectively). However, both Y640F and D661Y STAT3 mutant groups had significantly lower ANC compared to their WT counterparts (*P* = .0022 and .0009, respectively, Sidak comparison following one‐way ANOVA) (Figure 6C). This finding in homogeneous single STAT3 mutation sample groups was different from the Original Cohort, where a heterogeneous STAT3 mutant population did not differ from WT samples (Figure S2C). In summary, molecularly defined patients with either Y640F or D661Y STAT3 mutation had significantly lower ANC than WT STAT3 counterparts.

**FIGURE 7:**
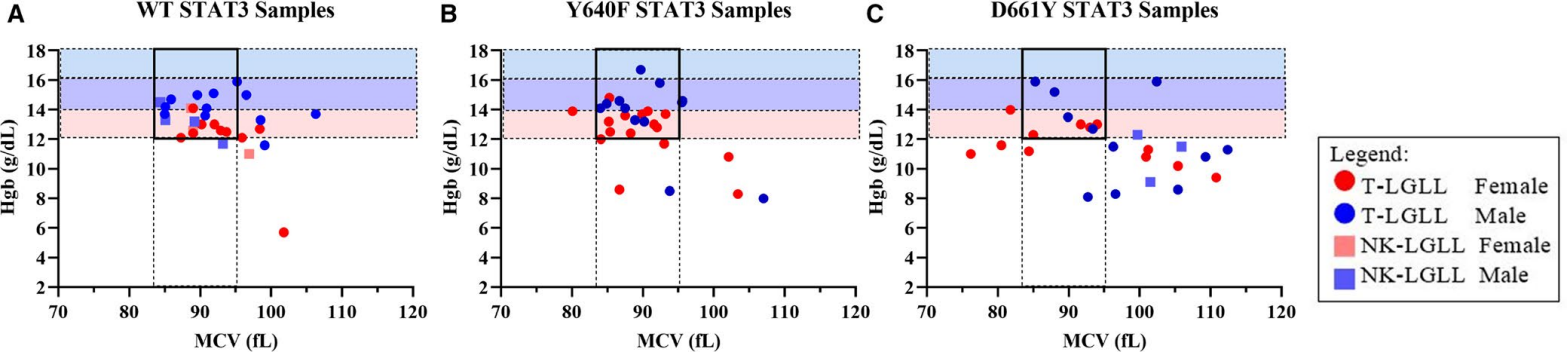
Mean corpuscular volume (MCV) vs hemoglobin (Hgb) from the Expanded Cohort according to STAT3 mutation status. MCV and Hgb values for each STAT3 mutation status group. Normal reference ranges are indicated by the dotted rectangles. MCV range (83.0‐95.0 fL) is indicated as a vertical rectangle. Hgb range (12‐16 g/dL for females and 14‐18 g/dL for males) is indicated as horizontal rectangles. The lower end for females and higher end for males are shown as pink and blue rectangles, respectively. Overlap between sexes is indicated as a purple rectangle. The square with the thick black border encompasses patients who had values inside the normal range for both MCV and Hgb. The patients who were outside both of the normal ranges were tallied, and a chi‐square test showed a significant difference (*P* = .015) between the groups. Refer to the legend for information on color and shape coding

We examined whether there was a relationship between MCV and Hgb (Figure 7). We found that 5 out of 29 WT samples (Figure 7A), 3 out of 28 Y640F samples (Figure 7B), and 11 out of 26 D661Y samples (Figure 7C) were outside the normal range for both MCV and Hgb. A chi‐square test indicated that this distribution was different from chance (*P* = .015). Taken together, the Expanded Cohort of approximately equal numbers of WT, Y640F, and D661Y STAT3 samples showed differing hematological parameters based on mutation and sex.

Finally, we conducted a follow‐up analysis on a subset of the WT, Y640F, and D661Y STAT3 mutation groups to determine any changes in MCV and Hgb values within these groups over time (Figure S4). The mean MCV was approximately the same in each STAT3 mutation group at both time points (*t* tests showed no significant difference), with the D661Y group showing a higher mean at both initial and follow‐up time points (Figure S4A,C,E). The Hgb average stayed the same in the WT group and improved slightly in the Y640F and D661Y groups although the change was not statistically significant (Figure S4B,D,F). In summary, we have shown that the elevated MCV and low Hgb seen in the D661Y samples persist over time and that MCV and Hgb are relatively stable in all mutational groups.

## 4 DISCUSSION

This study accomplished three goals: (a) completed the first study measuring serum sphingolipids in LGL leukemia, (b) compared the two LGL leukemia subtypes in one study to find biomarker differences between the two subtypes, and (c) established relationships between specific STAT3 mutations and clinical data. These results provide interesting directions for future biomarker studies and demonstrate that both sex and specific STAT3 mutations are associated with clinical symptoms.

Sphingolipid serum measurements have emerged as informative clinical parameters. Originally named after the Sphinx, indicating their mysterious nature,^29^ it is now clear that this class of lipids may be markers of pathogenesis in leukemia and cardiovascular disease^26^, ^27^, ^32^, ^41^ or even markers of treatment response.^42^ It was previously reported that dysregulated sphingolipid metabolism contributes to survival of leukemic LGL cells,^25^ but this current study was the first to measure serum sphingolipids in LGL leukemia patients. In this study, three sphingomyelins (SMC20, SMC22, SMC24) were significantly decreased in LGL leukemia samples compared to normal donors (Figure 2; Table S3). A study found increased levels of long‐chain sphingomyelins in the synovial fluid of both rheumatoid arthritis and osteoarthritis patients compared to normal donors.^43^ Synovial fluid contains lubricants like hyaluronan, and addition of exogenous compounds can disrupt its function.^44^ Since synovial fluid is considered an ultrafiltrate of blood plasma, it is plausible that excess sphingolipids, cytokines, or other macromolecules can accumulate in the synovial fluid. A diagnostic benefit of this relationship is that obtaining a blood (plasma) sample from a patient is much less invasive than synovial fluid, thus, blood biomarkers may serve as indicators of synovial fluid status.^45^ The similarities between LGL leukemia and rheumatoid arthritis in regards to chronic nature, symptoms, and treatment emphasizes the potential for sphingolipids as biomarkers.

Ceramides can be converted to sphingomyelin by the enzyme sphingomyelin synthase, and the reverse reaction is mediated by sphingomyelinase. Therefore, a delicate balance must be maintained in the cell to ensure equilibrium of these pools of sphingolipids.^32^ With the significantly lower sphingomyelin levels in LGL leukemia serum, ceramides should correspondingly be increased. Our data do not show significant elevation of ceramides in LGL leukemia patient serum, however, it is noticeable that most ceramide species in the LGL leukemia patients have a wider range and higher mean compared to the normal controls (Tables S2 and S3). This occurrence of altered sphingomyelin and ceramide serum levels was recently documented in a study of stage IV colorectal cancer patients.^46^ The ceramide increase in these cancer patients is puzzling since these sphingolipids typically have antiproliferative effects.^32^ However, increased ceramides in other cancers have been reported^47^, ^48^, ^49^, ^50^, ^51^ and it is possible that ceramides could have dual roles depending on the cellular context and chain length. In addition, serum lipidomics may not be identical to changes within the tumor cells. We found S1P, a pro‐survival sphingolipid, had a positive linear relationship with MCV in WT STAT3, but not mutant, patient samples (Figure 4D, top graph). While mutant STAT3 is thought to contribute to survival of LGL cells,^23^ perhaps in the absence of mutation, higher levels of S1P in WT samples contributes to LGL survival. Two sphingomyelins (SMC24 and SMC26:1) showed positive and linear relationships with WT and mutant STAT3, respectively (Figure 4D), but the role of these sphingolipids in LGL leukemia is not known. In addition, sex and fasting differences between male and female serum sphingolipid profiles in a cross‐sectional study have been reported,^52^ further underscoring how many factors can influence biomarker measurements. Importantly, because these variables were analyzed post hoc, a follow‐up hypothesis‐driven study should be conducted to ensure these were not false positive results.

While the role of glycosylated sphingolipids in LGL leukemia pathogenesis is currently unknown, a previous study found elevated glucosylceramide synthase expression in leukemic NK cells.^28^ Furthermore, glycosylated lipids are found in normal human bone marrow, with the majority of this species consisting of a dihexosylceramide.^53^ In this study, we observed that four hexosylceramide species had a positive and negative linear relationship with MCV in WT and mutant STAT3 samples, respectively (Figure 4C). The biological significance of this finding is unclear at this time and is something that could be investigated further. Clustering analysis showed a small group of LGL leukemia patients (box 5, Figure 2) had uniformly upregulated species of hexosylceramides, however, the potential biological relevance is unknown. A study in multiple sclerosis cerebrospinal fluid demonstrated elevated levels of HexC16 as a disease progression biomarker.^54^ Furthermore, additional species of hexosylceramides were elevated in serum or plasma of other neurodegenerative diseases.^55^ Since LGL leukemia often co‐occurs with and shares many features with autoimmune disease^1^, ^2^ it is possible that hexosylceramides could be a new class of biomarkers to predict symptoms and treatment necessity.

Our second goal was to compare the two LGL leukemia subtypes to normal donors in one study. Most previous serum cytokine studies have focused on one LGL leukemia subtype,^14^, ^15^, ^16^, ^17^, ^18^, ^19^, ^20^, ^21^, ^22^, ^23^ with just one measuring a limited subset of seven chemokines in both subtypes.^24^ Furthermore, we conducted rigorous multiple testing corrections, which has not been done in most other published serum studies in LGL leukemia. Therefore, we measured 24 cytokines in both LGL leukemia subtypes and compared these to normal donors. These 24 included those previously reported as significantly different in LGL leukemia compared to normal donors, with the intention of identifying unique changes that are specific to the subtypes of LGL leukemia. Furthermore, the serum sphingolipid measurements were completely novel. Comparisons between the subtypes showed only one difference, elevated C22 just short of significance (*P* = .060) in NK‐LGL serum compared to T‐LGL serum. Comparing LGL leukemia to normal donor serum, three cytokines were significantly different after rigorous multiple testing correction: EGF was decreased and IP‐10 and G‐CSF were increased in LGL leukemia serum compared to normal donors. These findings agree with a previous NK‐LGL leukemia study.^17^ Sources of EGF are salivary glands, duodenum, pancreas,^56^ and megakaryocytes.^57^ It is currently unclear why LGL leukemia patients would have lower serum EGF. One potential reason could be a damaged production site, as was suggested with similar results from inflammatory bowel disease patients.^58^ An assessment of tissue damage would need to be conducted to further explore this hypothesis. IP‐10, also called CXCL10, is a chemokine that functions to recruit lymphocytes.^13^ Three studies found elevated IP‐10 in LGL leukemia patient serum.^14^, ^17^, ^24^ The Savola et al study also reported elevated IP‐10 in Felty's syndrome, but not rheumatoid arthritis patients.^14^ Felty's syndrome is a disease that shares many features with LGL leukemia.^59^ However, a larger study of untreated early rheumatoid arthritis found elevated IP‐10 which correlated with disease activity.^60^ Therefore, these three similar diseases may have IP‐10 as a common marker of pathogenesis. G‐CSF is a cytokine involved in bone marrow stimulation.^13^ It would be reasonable to expect LGL leukemia patients to have elevated levels of G‐CSF if their bone marrow is working harder to produce blood cells.

Finally, the LGL Leukemia Registry enabled us to study large groups of the most common STAT3 mutations to discern whether different mutations affect blood counts. We compared WT STAT3 to single Y640F or D661Y STAT3 mutation groups as these are the most frequently occurring SH2 domain mutations reported in LGL leukemia.^5^, ^7^, ^18^, ^33^ Red blood cell parameters (MCV, Hgb, and Hct) showed significant differences in most of these clinical values, namely the male D661Y samples (Figure 5A‐D). Furthermore, neutropenia was observed in both the Y640F and D661Y groups (Figure 6C). Previous publications have shown that STAT3 mutations were associated with neutropenia, anemia, or both compared to STAT3 WT patients, but these were generally a heterogeneous mix of mutant samples rather than precise individual mutational groups as analyzed here.^5^, ^7^, ^10^, ^11^, ^18^, ^33^, ^34^, ^35^ In some cases, the role of STAT3 mutations seems to be dependent on ethnicity, such as those that co‐occur with PRCA.^10^, ^34^, ^35^ Sex distinctions according to STAT3 mutation subtypes were not made in any of these publications, but our work indicates that this could be another factor to consider when analyzing LGL leukemia patient clinical features.

Most STAT3 mutations in the SH2 domain create a hydrophobic change in the amino acid.^7^, ^61^ The Y640F mutant can homodimerize independently of cytokine stimulation, and thus, carry out transcriptional activities,^62^ however, it is likely that in most cases mutants still depend on upstream cytokine stimulation.^63^ In the Original Cohort, the five highest data points were all D661 mutations, with four changed to tyrosine (polar side chain with phosphorylation capability) and one changed to isoleucine (hydrophobic side chain) (Figure 4A). Therefore, the mechanism of mutant STAT3 activation is not clear and a larger group of D661 mutations (other than tyrosine) would need to be characterized.

The purpose of the Expanded Cohort was to substantiate the association of D661Y mutation with macrocytic anemia observed in the Original Cohort (Figure 5A). Since Hgb tended to be low in this mutant population, we plotted MCV and Hgb based on STAT3 mutation status (Figure 7) and found that the D661Y mutant group had a much greater chance of having simultaneously low Hgb and high MCV, compared to WT or Y640F patients. This suggests that STAT3 mutation status could be another factor for predicting or treating anemia. The occurrence of low Hgb and high MCV has been reported in two case studies of T‐LGL leukemia and an associated disease, but not in the context of STAT3 mutation status.^64^, ^65^ A previous study found that STAT3 SH2 domain mutation is correlated with smaller granular lymphocytes in T‐LGL leukemia.^66^ In particular, a small subset of four patients with unusually small mean lymphocyte diameter all had D661Y mutation (one was Y640F/D661Y) with anemia (two of them were diagnosed with PRCA).^66^

Anecdotally, it has been stated that LGL leukemia patients tend to have macrocytic anemia.^37^ One study reported macrocytosis (MCV > 101 fL) in 23% of a 68‐patient cohort,^36^ but this is the first LGL leukemia study to report individual MCV values and to differentiate based on sex and STAT3 mutation status. In our Expanded Cohort of 83 patients, we found an incidence of 32.5% with MCV > 95 fL, with 16.9% of them >101 fL. When divided by mutation, STAT3 WT incidence was 31.0% >95 fL (with 6.9% >101 fL), Y640F incidence was 17.9% >95 fL (with 10.7% >101 fL), and D661Y incidence was 50% >95 fL (with 34.6% >101 fL). The general population incidence of macrocytosis was reported as 3% in a Finnish study, with the most common cause as alcoholism.^67^ A review of the 14 patients who had MCV > 101 fL (those with the highest values) did not find evidence of concomitant myelodysplastic syndrome (MDS), which can cause elevated MCV. Taken together, the incidence of macrocytic anemia in LGL leukemia patients is higher than the general population and particularly higher in patients with a D661Y STAT3 mutation. Occurrence of macrocytic anemia is generally unexplained in LGL leukemia. It is also unknown at this time why patients with the D661Y STAT3 mutation would have a higher incidence. This warrants further studies to understand the mechanism and if the mutation could be a useful prognostic marker.

We conducted a follow‐up analysis on a subset of the Expanded Cohort to evaluate natural progression of the disease in groups with defined STAT3 molecular status. Interestingly, we found that the groups had very similar MCV and Hgb average values at follow‐up. The D661Y group exhibited a higher MCV mean compared to the other groups at both time points. While this follow‐up is quite informative and interesting, it should be validated within a prospective study containing uniform time points.

It is unclear at this time why only male D661Y patients have low Hgb and macrocytic anemia. However, two separate studies provide some data to support our sex difference observations. One study measured a large number of macromolecules in normal donor male and female serum samples and found that certain cytokines common to our study were elevated in females but not males and vice versa.^68^ Furthermore, another study found that a conditional epithelial cell STAT3 knockout in mouse models of K‐ras mutant lung cancer resulted in different tumor burdens between male and female mice.^69^ Finally, a previous prospective study determined patients with Y640F mutation were more likely to respond to methotrexate.^15^ Therefore, building on this information, it is possible that examining clinical presentation and treatment outcomes in patients might be even more insightful if grouped by both STAT3 mutation status and sex. In particular, previous work has suggested Y640F and D661Y STAT3 mutations are both activating,^7^ therefore, the difference in phenotype we observe here is unexpected. This also suggests that STAT3 mutations should not be grouped together when analyzing clinical data and that biochemical characterization of individual mutations could be very informative.

In summary, this study measured serum levels of cytokines and sphingolipids in T‐and NK‐LGL leukemia. We found three cytokines and four sphingolipids were significantly different in LGL leukemia compared to normal donor samples. We also observed interesting relationships between some cytokines and sphingolipids with MCV, dependent on STAT3 mutation status. We expanded the cohort to examine single Y640F or D661Y STAT3 mutation samples compared to WT samples. We found that male D661Y samples tended to have elevated MCV and low Hgb, while both Y640F and D661Y samples were neutropenic in both sexes. These data provide exciting new directions for research such as sphingolipids as prognostic measurements or their synthetic enzymes as new drug targets. In addition, careful analysis of STAT3 mutation type indicated that particular mutations may differentially contribute to disease manifestation.

## CONFLICT OF INTEREST

TPL is on the scientific advisory board of BIONIZ Therapeutics, Keystone Nano, Dren Bio, and Kymera Therapeutics. There are no conflicts with the results presented in this manuscript.

## AUTHOR CONTRIBUTIONS

KCO participated in research design, conducted experiments, collected data, performed data analysis, and wrote the manuscript. KBM participated in research design, collected data, performed data analysis and contributed to the writing of the manuscript. MKJ performed data analysis and contributed to the writing of the manuscript. PMKL participated in research design, conducted experiments, collected data, and contributed to the writing of the manuscript. TLO participated in research design, collected data, and performed data analysis. MFT collected data and performed data analysis. TEF conducted experiments, performed data analysis, and contributed to the writing of the manuscript. DJF participated in research design, performed data analysis, and contributed to the writing of the manuscript. TPL participated in research design, performed data analysis, and contributed to the writing of the manuscript.

## Supporting information

Fig S1

Fig S2

Fig S3

Fig S4

Table S1

Table S2

Table S3

Table S4

Table S5

## Data Availability

The data that support the findings of this study are available from the corresponding author upon reasonable request.
