# Supplementary figures and images for: Large granular lymphocyte leukemia serum and corresponding hematological parameters reveal unique cytokine and sphingolipid biomarkers and associations with STAT3 mutations
Source: Cancer Med. 2020 Jul 25;9(18):6533–49. doi: 10.1002/cam4.3246 (PMC7520360; doi:10.1002/cam4.3246)

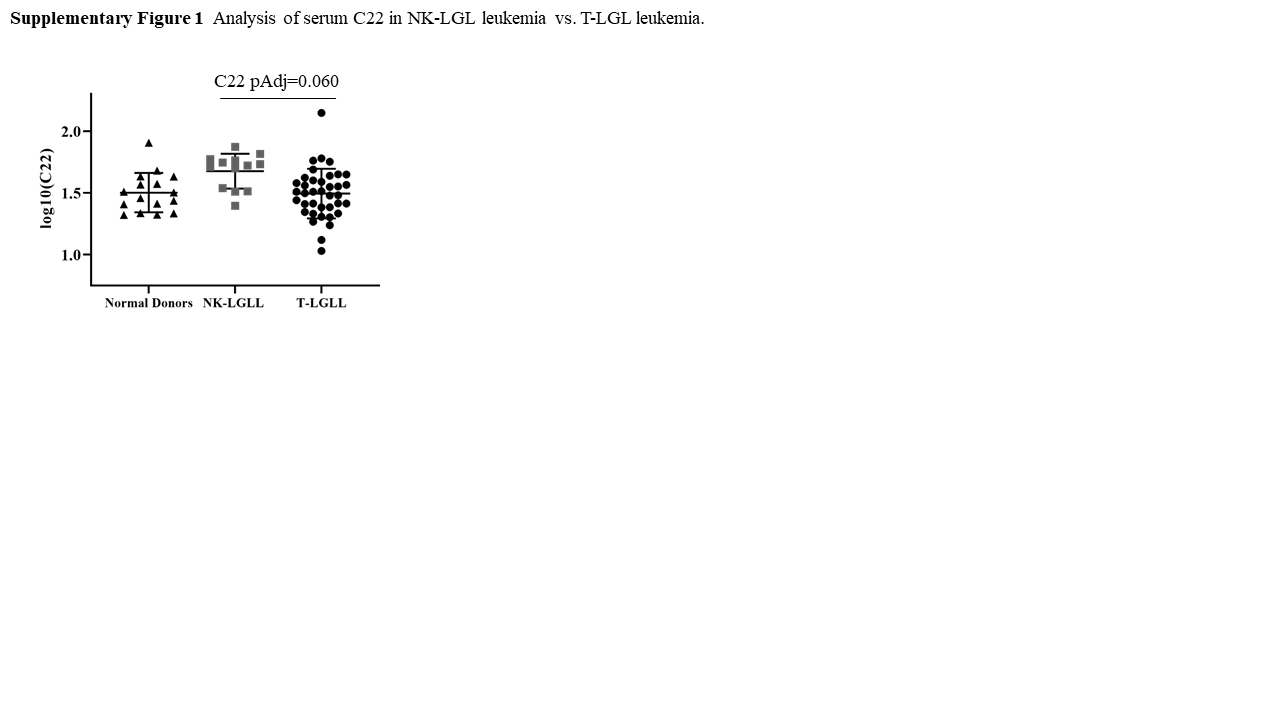

Supplement: Supplementary file 1 — Fig S1 [file CAM4-9-6533-s001.tif]

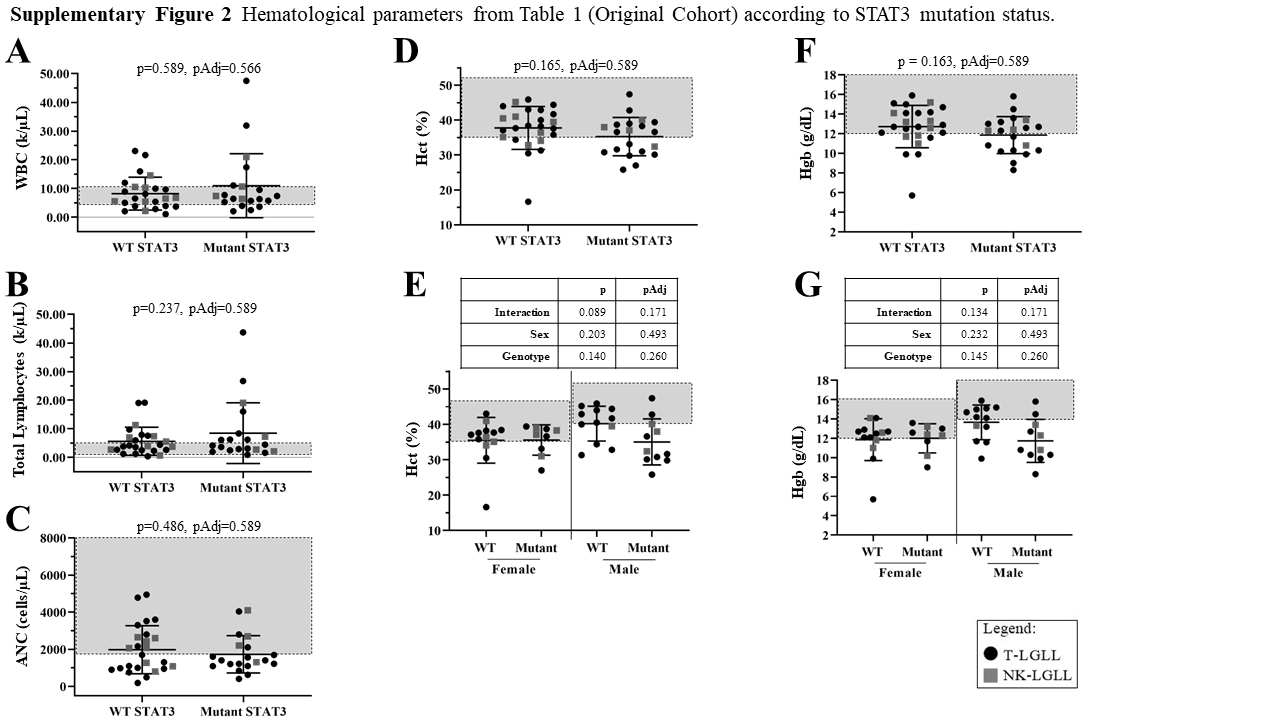

Supplement: Supplementary file 2 — Fig S2 [file CAM4-9-6533-s002.tif]

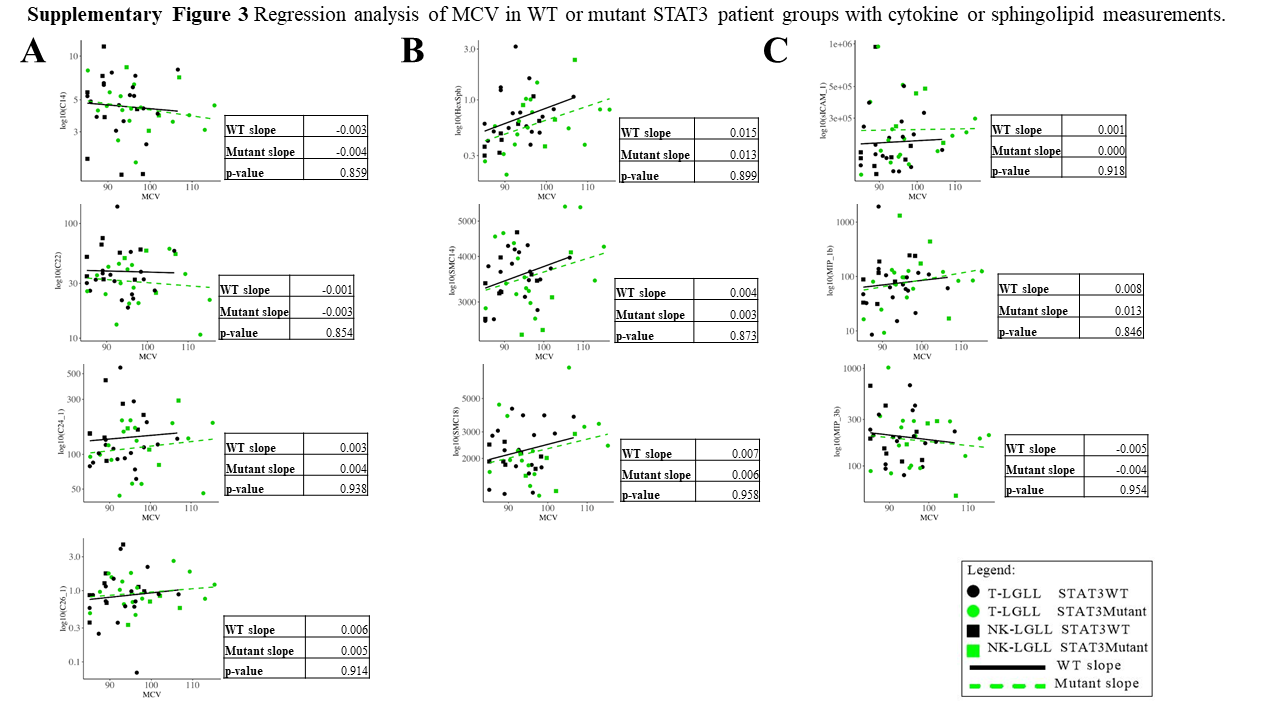

Supplement: Supplementary file 3 — Fig S3 [file CAM4-9-6533-s003.tif]

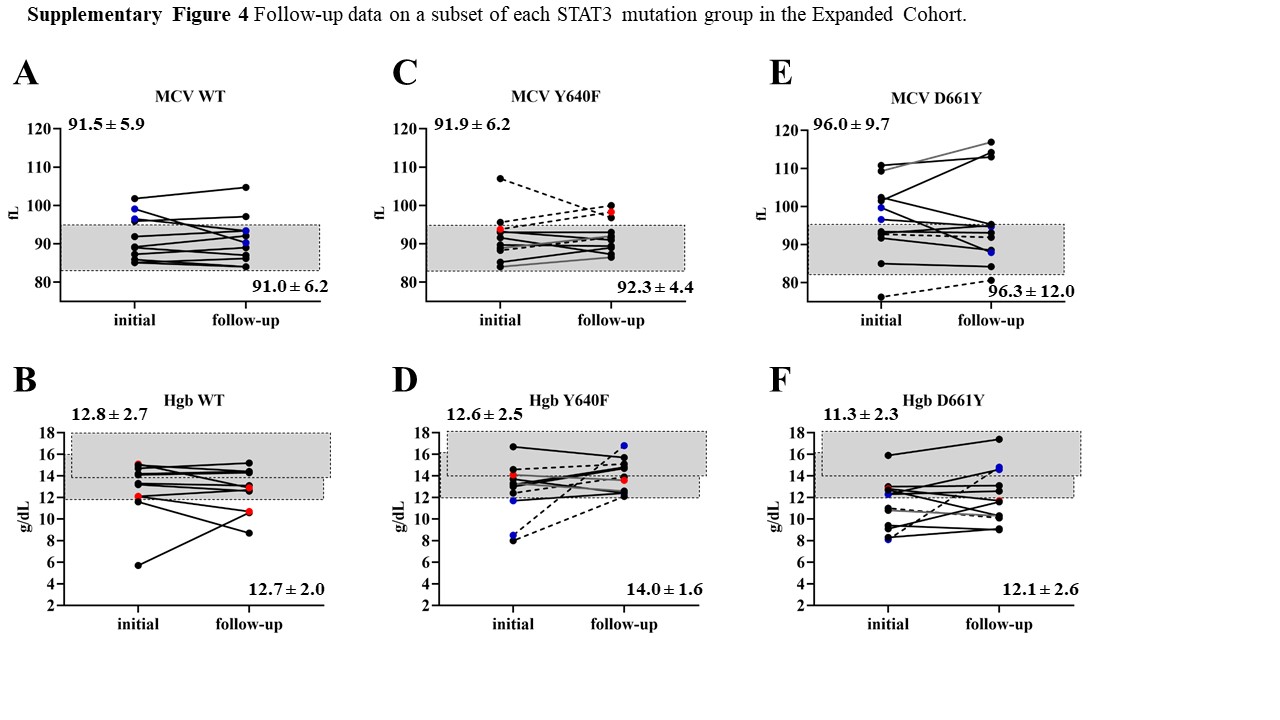

Supplement: Supplementary file 4 — Fig S4 [file CAM4-9-6533-s004.jpg]
