# Supplementary material for: Large granular lymphocyte leukemia serum and corresponding hematological parameters reveal unique cytokine and sphingolipid biomarkers and associations with STAT3 mutations
Source: Cancer Med. 2020 Jul 25;9(18):6533–49. doi: 10.1002/cam4.3246 (PMC7520360; doi:10.1002/cam4.3246)
Supplement: Supplementary file 5 — Table S1 [file CAM4-9-6533-s005.docx]

**Supplementary Table 1. Serum cytokine measurements in LGL leukemia patients and normal donors.** The cytokine measurements for the LGL leukemia (Original Cohort, n=13 NK and n=37 T) and normal donor (n=16) samples are shown as mean, SD, minimum and maximum values. Underlined cytokines indicate a significant p-value after multiple testing correction (p-values in **Supplementary Table 3**). All values are reported as pg/mL. In some cases, the values exceeded the limit of detection of the Luminex Multiplex assay. Cytokines at the maximum limits were: IFN-γ = 1.7 pg/mL, IL-18 = 2885 pg/mL, IP-10 = 8610 pg/mL, and MIG = 48748 pg/mL. Cytokines at the minimum limits were: G-CSF = 2.3 pg/mL, FasL = 50.2 pg/mL, Flt3L = 2.7 pg/mL, IFNa2 = 9.7 pg/mL, IL-1RA = 2.8 pg/mL, IL-6 = 2.4 pg/mL, IL-18 = 8.6 pg/mL, IL-10 = 1.4 pg/mL, and TRAIL = 2.3 pg/mL. Four cytokines with ^ notation means n=15 normal donors due to instrument failure.

|  |  |  | **Cytokines (pg/mL)** | | | | | | | | | | | | |
| --- | --- | --- | --- | --- | --- | --- | --- | --- | --- | --- | --- | --- | --- | --- | --- |
|  |  |  | **MIG** | **IP-10** | **EGF** | **G-CSF** | **Flt-3 Lig.** | **IFNa2** | **IFNg** | **MIP-3b** | **IL-10** | **IL-1RA** | **TRAIL** | **IL-6** | **IL-8** |
|  | **Normal Donors** | **Mean** | 1272 | 234.7 | 1006.7 | 9.6 | 13.4 | 11.4 | 11.3 | 172.6 | 10.5 | 70.9 | 2.3 | 7.9 | 400.3 |
|  |  | **SD** | 1614 | 88.1 | 625.2 | 22.5 | 24.8 | 6.5 | 8.6 | 82.4 | 19.7 | 60.6 | 0.0 | 11.7 | 425.0 |
|  |  | **Min** | 329 | 135.3 | 105.5 | 2.3 | 2.7 | 9.8 | 1.8 | 27.0 | 1.4 | 3.5 | 2.3 | 2.4 | 38.3 |
|  |  | **Max** | 6928 | 460.8 | 2172.0 | 92.8 | 102.7 | 35.9 | 31.8 | 370.5 | 70.6 | 197.5 | 2.3 | 46.7 | 1309.0 |
|  |  |  |  |  |  |  |  |  |  |  |  |  |  |  |  |
|  | **NK-LGLL** | **Mean** | 9669 | 1579.4 | 240.4 | 115.5 | 93.5 | 102.8 | 66.1 | 236.4 | 17.6 | 135.8 | 4.8 | 52.5 | 402.6 |
|  |  | **SD** | 15459 | 2223.2 | 137.5 | 186.2 | 134.9 | 155.5 | 107.5 | 161.3 | 23.3 | 269.5 | 3.3 | 89.8 | 864.3 |
|  |  | **Min** | 438 | 334.5 | 65.9 | 4.8 | 2.7 | 9.8 | 1.8 | 49.6 | 1.4 | 4.0 | 2.3 | 2.4 | 4.0 |
|  |  | **Max** | 48748 | 8610.0 | 555.7 | 497.4 | 381.9 | 467.1 | 300.5 | 663.8 | 74.7 | 1003.0 | 10.8 | 268.9 | 2885.0 |
|  |  |  |  |  |  |  |  |  |  |  |  |  |  |  |  |
|  | **T-LGLL** | **Mean** | 8050 | 989.0 | 266.4 | 80.6 | 38.0 | 40.3 | 30.5 | 234.3 | 7.5 | 100.5 | 11.2 | 104.1 | 273.2 |
|  |  | **SD** | 15029 | 1439.4 | 176.0 | 151.4 | 83.3 | 63.2 | 42.3 | 177.9 | 11.5 | 308.8 | 27.8 | 555.7 | 626.9 |
|  |  | **Min** | 398 | 190.5 | 41.3 | 2.3 | 2.7 | 9.8 | 1.7 | 80.4 | 1.4 | 2.8 | 2.3 | 2.4 | 5.7 |
|  |  | **Max** | 48748 | 8610.0 | 726.7 | 833.8 | 410.3 | 365.8 | 165.3 | 1019.0 | 49.5 | 1880.0 | 160.2 | 3390.0 | 2885.0 |

|  |  |  | **Cytokines (pg/mL)** | | | | | | | | | | |
| --- | --- | --- | --- | --- | --- | --- | --- | --- | --- | --- | --- | --- | --- |
|  |  |  | **SDF-1a+b** | **MIP-1b** | **IL-18** | **Eotaxin 2** | **RANTES** | **sICAM-1^** | **sFas Lig.^** | **sFas ^** | **sVCAM-1 ^** | **TGFB1** | **TGFB2** |
|  | **Normal Donors** | **Mean** | 1475 | 61.8 | 15.8 | 256.2 | 92671 | 182105 | 83.0 | 6170 | 811423 | 41565 | 3206 |
|  |  | **SD** | 677 | 24.8 | 7.1 | 36.1 | 35546 | 61702 | 37.4 | 1493 | 190555 | 14351 | 987 |
|  |  | **Min** | 226 | 7.0 | 8.6 | 205.9 | 43849 | 116765 | 50.3 | 4055 | 405349 | 20547 | 1800 |
|  |  | **Max** | 2936 | 121.0 | 36.7 | 337.4 | 152042 | 300248 | 190.8 | 10055 | 1143501 | 70562 | 5261 |
|  |  |  |  |  |  |  |  |  |  |  |  |  |  |
|  | **NK-LGLL** | **Mean** | 2222 | 254.8 | 54.8 | 264.1 | 74715 | 284117 | 260.0 | 8529 | 1444842 | 37848 | 2564 |
|  |  | **SD** | 1289 | 343.7 | 42.9 | 125.4 | 36658 | 230604 | 260.5 | 2748 | 699267 | 14786 | 845 |
|  |  | **Min** | 1013 | 16.9 | 8.6 | 78.0 | 26508 | 121444 | 50.3 | 3379 | 746495 | 15976 | 949 |
|  |  | **Max** | 5166 | 1319.0 | 157.5 | 525.5 | 167384 | 951802 | 807.2 | 13058 | 3033928 | 74116 | 4238 |
|  |  |  |  |  |  |  |  |  |  |  |  |  |  |
|  | **T-LGLL** | **Mean** | 1943 | 129.0 | 47.3 | 355.3 | 71959 | 242058 | 140.8 | 7400 | 1268913 | 29344 | 2172 |
|  |  | **SD** | 735 | 309.5 | 87.0 | 147.6 | 37028 | 154243 | 110.0 | 3154 | 752074 | 12865 | 936 |
|  |  | **Min** | 439 | 8.5 | 8.6 | 137.2 | 9684 | 120175 | 50.3 | 2098 | 45386 | 9020 | 781 |
|  |  | **Max** | 3860 | 1932.0 | 524.1 | 943.6 | 164904 | 956219 | 476.5 | 16048 | 3846121 | 56820 | 4404 |
