# Supplementary material for: Large granular lymphocyte leukemia serum and corresponding hematological parameters reveal unique cytokine and sphingolipid biomarkers and associations with STAT3 mutations
Source: Cancer Med. 2020 Jul 25;9(18):6533–49. doi: 10.1002/cam4.3246 (PMC7520360; doi:10.1002/cam4.3246)
Supplement: Supplementary file 6 — Table S2 [file CAM4-9-6533-s006.docx]

**Supplementary Table 2.** **Serum sphingolipid measurements in LGL leukemia patients and normal donors.** The sphingolipid measurements for the LGL leukemia (Original cohort, n=13 NK and n=37 T) and normal donor (n=16) samples are shown as mean, SD, minimum and maximum values. Underlined sphingolipids indicate a significant p-value after multiple testing correction (p-values in **Supplementary Table 3**). All values are reported as pmol/mL serum.

|  |  |  | **Ceramides (pmol/mL serum)** | | | | | | | | | **Hexosylceramides (pmol/mL serum)** | | | | | | | | |
| --- | --- | --- | --- | --- | --- | --- | --- | --- | --- | --- | --- | --- | --- | --- | --- | --- | --- | --- | --- | --- |
|  |  |  | **C14** | **C16** | **C18** | **C20** | **C22** | **C24** | **C24:1** | **C26** | **C26:1** | **HexC14** | **HexC16** | **HexC18** | **HexC20** | **HexC22** | **HexC24** | **HexC24:1** | **HexC26** | **HexC26:1** |
|  | **Normal Donors** | **Mean** | 5.8 | 44.3 | 13.7 | 8.0 | 34.0 | 45.3 | 98.4 | 3.8 | 1.1 | 3.8 | 55.5 | 2.4 | 2.6 | 16.5 | 10.1 | 30.9 | 0.019 | 0.124 |
|  |  | **SD** | 1.4 | 12.1 | 6.1 | 2.5 | 15.0 | 12.6 | 46.9 | 1.0 | 0.7 | 0.9 | 15.4 | 0.6 | 0.7 | 5.6 | 3.2 | 9.9 | 0.016 | 0.076 |
|  |  | **Min** | 3.8 | 25.5 | 4.5 | 2.8 | 20.9 | 26.6 | 31.5 | 2.5 | 0.3 | 2.4 | 23.9 | 1.6 | 1.8 | 9.3 | 5.4 | 18.3 | 0.002 | 0.017 |
|  |  | **Max** | 8.7 | 66.5 | 26.4 | 11.8 | 80.3 | 71.2 | 182.7 | 5.3 | 3.0 | 5.2 | 74.8 | 4.2 | 4.4 | 28.9 | 16.2 | 48.7 | 0.057 | 0.276 |
|  |  |  |  |  |  |  |  |  |  |  |  |  |  |  |  |  |  |  |  |  |
|  | **NK-LGLL** | **Mean** | 5.1 | 72.8 | 22.7 | 14.1 | 49.6 | 59.7 | 195.7 | 3.3 | 1.1 | 4.5 | 77.3 | 2.3 | 2.6 | 15.5 | 10.9 | 41.1 | 0.017 | 0.173 |
|  |  | **SD** | 3.0 | 49.3 | 22.7 | 10.3 | 14.5 | 23.0 | 100.5 | 1.3 | 1.1 | 2.8 | 41.3 | 1.1 | 0.9 | 6.1 | 5.5 | 18.9 | 0.016 | 0.109 |
|  |  | **Min** | 1.5 | 32.4 | 9.0 | 4.1 | 24.8 | 24.1 | 81.0 | 0.7 | 0.3 | 1.3 | 36.4 | 1.0 | 1.2 | 7.0 | 4.8 | 18.0 | 0.003 | 0.024 |
|  |  | **Max** | 11.6 | 226.8 | 93.9 | 45.7 | 74.4 | 117.0 | 438.8 | 5.1 | 4.4 | 11.8 | 176.9 | 4.9 | 4.1 | 25.6 | 22.1 | 85.7 | 0.063 | 0.401 |
|  |  |  |  |  |  |  |  |  |  |  |  |  |  |  |  |  |  |  |  |  |
|  | **T-LGLL** | **Mean** | 4.7 | 46.6 | 15.3 | 8.9 | 34.8 | 47.0 | 131.1 | 3.5 | 1.1 | 4.3 | 66.6 | 2.1 | 2.7 | 16.1 | 11.1 | 43.3 | 0.033 | 0.198 |
|  |  | **SD** | 1.6 | 17.2 | 5.9 | 5.9 | 21.4 | 45.1 | 89.5 | 1.3 | 0.7 | 2.9 | 41.6 | 0.9 | 1.5 | 10.1 | 10.9 | 32.8 | 0.088 | 0.228 |
|  |  | **Min** | 1.8 | 23.3 | 4.8 | 2.9 | 10.7 | 15.1 | 43.8 | 1.2 | 0.1 | 1.8 | 29.5 | 0.7 | 0.8 | 5.7 | 3.3 | 13.2 | 0.003 | 0.002 |
|  |  | **Max** | 8.1 | 99.3 | 30.3 | 40.0 | 140.2 | 295.7 | 564.8 | 7.5 | 3.9 | 20.2 | 276.9 | 5.7 | 8.8 | 60.9 | 69.9 | 176.4 | 0.546 | 1.339 |

|  |  |  | **Long Chain Bases (pmol/mL serum)** | | | | | | **Sphingomyelins (pmol/mL serum)** | | | | | | | | |
| --- | --- | --- | --- | --- | --- | --- | --- | --- | --- | --- | --- | --- | --- | --- | --- | --- | --- |
|  |  |  | **Sph** | **dhSph** | **S1P** | **dhS1P** | **HexSph** | **LysoSM** | **SMC14** | **SMC16** | **SMC18** | **SMC20** | **SMC22** | **SMC24** | **SMC24:1** | **SMC26** | **SMC26:1** |
|  | **Normal Donors** | **Mean** | 87.6 | 16.4 | 864 | 141.0 | 0.7 | 0.6 | 4040 | 30197 | 3151 | 1747 | 1476 | 399.2 | 4140 | 13.6 | 10.3 |
|  |  | **SD** | 27.3 | 6.2 | 220 | 37.8 | 0.3 | 0.5 | 520 | 3811 | 721 | 593 | 599 | 162.4 | 1184 | 2.7 | 3.0 |
|  |  | **Min** | 33.0 | 8.0 | 534 | 67.0 | 0.3 | 0.1 | 3305 | 22224 | 1660 | 1062 | 736 | 183.5 | 2426 | 8.8 | 6.2 |
|  |  | **Max** | 143.3 | 33.7 | 1350 | 223.5 | 1.3 | 2.4 | 4971 | 36176 | 4262 | 2954 | 2662 | 657.6 | 6853 | 17.6 | 16.4 |
|  |  |  |  |  |  |  |  |  |  |  |  |  |  |  |  |  |  |
|  | **NK-LGLL** | **Mean** | 85.4 | 18.7 | 828 | 155.4 | 0.7 | 0.9 | 3410 | 26467 | 2238 | 1068 | 792 | 217.9 | 2703 | 13.9 | 7.9 |
|  |  | **SD** | 68.0 | 14.3 | 250 | 84.7 | 0.6 | 0.4 | 667 | 10456 | 891 | 473 | 394 | 116.5 | 1148 | 8.0 | 3.9 |
|  |  | **Min** | 18.8 | 4.4 | 521 | 62.3 | 0.3 | 0.4 | 2441 | 14695 | 1213 | 519 | 296 | 78.3 | 946 | 2.9 | 2.9 |
|  |  | **Max** | 222.9 | 48.0 | 1381 | 324.1 | 2.4 | 1.9 | 4659 | 42663 | 4804 | 2344 | 1703 | 531.5 | 4839 | 29.3 | 16.8 |
|  |  |  |  |  |  |  |  |  |  |  |  |  |  |  |  |  |  |
|  | **T-LGLL** | **Mean** | 78.2 | 13.7 | 933 | 144.6 | 0.8 | 1.0 | 3653 | 28903 | 2584 | 1097 | 861 | 234.4 | 3154 | 14.7 | 7.8 |
|  |  | **SD** | 53.1 | 10.9 | 274 | 57.5 | 0.5 | 0.5 | 693 | 12656 | 1337 | 578 | 484 | 107.5 | 1689 | 6.0 | 3.5 |
|  |  | **Min** | 26.9 | 2.1 | 394 | 51.1 | 0.2 | 0.3 | 2663 | 10576 | 1129 | 402 | 305 | 84.8 | 776 | 6.3 | 2.6 |
|  |  | **Max** | 278.6 | 60.3 | 1592 | 291.3 | 3.2 | 2.1 | 5482 | 59174 | 8035 | 2888 | 2343 | 545.5 | 7656 | 30.3 | 16.0 |
