# Supplementary material for: Large granular lymphocyte leukemia serum and corresponding hematological parameters reveal unique cytokine and sphingolipid biomarkers and associations with STAT3 mutations
Source: Cancer Med. 2020 Jul 25;9(18):6533–49. doi: 10.1002/cam4.3246 (PMC7520360; doi:10.1002/cam4.3246)
Supplement: Supplementary file 7 — Table S3 [file CAM4-9-6533-s007.docx]

**Supplementary Table 3. Statistical analysis of serum cytokine and sphingolipid levels in LGL leukemia vs. normal donor samples.** Statistical analyses with one-way ANOVA followed by multiple testing correction showed that seven serum biomarkers (in bold: EGF, IP-10, G-CSF, SMC22, SMC24, SMC20, and LysoSM) of the 57 measured were significantly different between NK-LGL leukemia (n=13), T-LGL (n=37) leukemia, and normal donor (n=16) serum. Global p-values before and after multiple testing correction are reported.

| **Cytokine or Lipid** | **p-value** | **Holm-Sidak adjusted p-value** | **F-statistic** | **df_num_** | **df_denom_** |
| --- | --- | --- | --- | --- | --- |
| **EGF** | **1.66E-06** | **9.45E-05** | 16.6 | 3 | 63 |
| **IP-10** | **3.69E-06** | **2.06E-04** | 15.4 | 3 | 63 |
| **G-CSF** | **1.64E-04** | **0.009** | 10.0 | 3 | 63 |
| **SMC22** | **1.91E-04** | **0.010** | 9.8 | 3 | 63 |
| **SMC24** | **2.01E-04** | **0.011** | 9.8 | 3 | 63 |
| **SMC20** | **2.95E-04** | **0.015** | 9.3 | 3 | 63 |
| **LysoSM** | **4.11E-04** | **0.021** | 8.8 | 3 | 63 |
| **C24:1** | **0.002** | 0.116 | 6.7 | 3 | 63 |
| **TGFB2** | **0.003** | 0.138 | 6.5 | 3 | 63 |
| **C16** | **0.004** | 0.209 | 5.9 | 3 | 63 |
| **IFNa2** | **0.004** | 0.211 | 5.9 | 3 | 63 |
| **MIG** | **0.006** | 0.255 | 5.6 | 3 | 63 |
| **Eotaxin-2** | **0.007** | 0.309 | 5.4 | 3 | 63 |
| **TGFB1** | **0.007** | 0.312 | 5.4 | 3 | 63 |
| **IL-8** | **0.007** | 0.322 | 5.3 | 3 | 63 |
| **C22** | **0.008** | 0.325 | 5.3 | 3 | 63 |
| **C20** | **0.014** | 0.594 | 4.5 | 3 | 63 |
| **IL-18** | **0.018** | 0.702 | 4.3 | 3 | 63 |
| **SMC24:1** | **0.018** | 0.703 | 4.3 | 3 | 63 |
| **SMC14** | **0.022** | 0.853 | 4.0 | 3 | 63 |
| **SMC18** | **0.025** | 0.910 | 3.9 | 3 | 63 |
| **sFas Ligand** | **0.026** | 0.935 | 3.9 | 3 | 63 |
| **MIP-1b** | **0.031** | 1 | 3.7 | 3 | 63 |
| **SMC26:1** | **0.034** | 1 | 3.6 | 3 | 63 |
| **IL-10** | **0.042** | 1 | 3.3 | 3 | 63 |
| **SDF-1a+b** | **0.043** | 1 | 3.3 | 3 | 63 |
| C24 | 0.062 | 1 | 2.9 | 3 | 63 |
| TRAIL | 0.071 | 1 | 2.8 | 3 | 63 |
| sVCAM-1 | 0.074 | 1 | 2.7 | 3 | 63 |
| C14 | 0.115 | 1 | 2.2 | 3 | 63 |
| Flt-3 Ligand | 0.124 | 1 | 2.2 | 3 | 63 |
| RANTES | 0.126 | 1 | 2.1 | 3 | 63 |
| dhSph | 0.134 | 1 | 2.1 | 3 | 63 |
| sICAM-1 | 0.157 | 1 | 1.9 | 3 | 63 |
| sFas | 0.158 | 1 | 1.9 | 3 | 63 |
| IL-6 | 0.163 | 1 | 1.9 | 3 | 63 |
| C18 | 0.167 | 1 | 1.8 | 3 | 63 |
| HexC16 | 0.238 | 1 | 1.5 | 3 | 63 |
| IL-1RA | 0.241 | 1 | 1.5 | 3 | 63 |
| HexC24:1 | 0.288 | 1 | 1.3 | 3 | 63 |
| C26 | 0.318 | 1 | 1.2 | 3 | 63 |
| SMC16 | 0.348 | 1 | 1.1 | 3 | 63 |
| MIP-3b | 0.351 | 1 | 1.1 | 3 | 63 |
| Sph | 0.368 | 1 | 1.0 | 3 | 63 |
| HexC18 | 0.391 | 1 | 1.0 | 3 | 63 |
| IFNg | 0.400 | 1 | 0.9 | 3 | 63 |
| S1P | 0.452 | 1 | 0.8 | 3 | 63 |
| SMC26 | 0.561 | 1 | 0.6 | 3 | 63 |
| HexC26:1 | 0.610 | 1 | 0.5 | 3 | 63 |
| HexC22 | 0.724 | 1 | 0.3 | 3 | 63 |
| HexSph | 0.781 | 1 | 0.2 | 3 | 63 |
| HexC24 | 0.843 | 1 | 0.2 | 3 | 63 |
| HexC26 | 0.859 | 1 | 0.2 | 3 | 63 |
| C26:1 | 0.917 | 1 | 0.1 | 3 | 63 |
| HexC14 | 0.963 | 1 | 0.04 | 3 | 63 |
| dhS1P | 0.963 | 1 | 0.04 | 3 | 63 |
| HexC20 | 0.972 | 1 | 0.03 | 3 | 63 |
