# Supplementary material for: Large granular lymphocyte leukemia serum and corresponding hematological parameters reveal unique cytokine and sphingolipid biomarkers and associations with STAT3 mutations
Source: Cancer Med. 2020 Jul 25;9(18):6533–49. doi: 10.1002/cam4.3246 (PMC7520360; doi:10.1002/cam4.3246)
Supplement: Supplementary file 8 — Table S4 [file CAM4-9-6533-s008.docx]

**Supplementary Table 4. Statistical analysis of cytokines and sphingolipids in T-LGL vs. NK- leukemia.** T-tests with Holm-Sidak multiple testing correction were performed to compare the two LGL leukemia subtypes. After multiple testing correction, no parameters were significant but C22 trended toward significance. Bold lettering indicates significance before multiple testing correction.

| **Cytokine or Lipid** | **p-value** | **Holm-Sidak adjusted p-value** | **T-statistic** | **df** |
| --- | --- | --- | --- | --- |
| **C22** | **0.001** | 0.060 | 5.3 | 3 |
| **C24:1** | **0.014** | 0.765 | 6.7 | 3 |
| **C24** | **0.017** | 0.935 | 2.9 | 3 |
| MIG | 0.444 | 1 | 5.6 | 3 |
| IP-10 | 0.207 | 1 | 15.4 | 3 |
| EGF | 0.932 | 1 | 16.6 | 3 |
| G-CSF | 0.755 | 1 | 10.0 | 3 |
| Flt-3 Ligand | 0.281 | 1 | 2.2 | 3 |
| IFNa2 | 0.276 | 1 | 5.9 | 3 |
| IFNg | 0.675 | 1 | 0.9 | 3 |
| MIP-3b | 0.993 | 1 | 1.1 | 3 |
| IL-10 | 0.030 | 1 | 3.3 | 3 |
| IL-1RA | 0.280 | 1 | 1.5 | 3 |
| TRAIL | 0.843 | 1 | 2.8 | 3 |
| IL-6 | 0.344 | 1 | 1.9 | 3 |
| IL-8 | 0.764 | 1 | 5.3 | 3 |
| SDF-1ab | 0.551 | 1 | 3.3 | 3 |
| MIP-1b | 0.069 | 1 | 3.7 | 3 |
| IL-18 | 0.177 | 1 | 4.3 | 3 |
| Eotaxin-2 | 0.044 | 1 | 5.4 | 3 |
| RANTES | 0.655 | 1 | 2.1 | 3 |
| sICAM-1 | 0.666 | 1 | 1.9 | 3 |
| sFas_Ligand | 0.193 | 1 | 3.9 | 3 |
| sFas | 0.150 | 1 | 1.9 | 3 |
| sVCAM-1 | 0.181 | 1 | 2.7 | 3 |
| TGFB1 | 0.046 | 1 | 5.4 | 3 |
| TGFB2 | 0.130 | 1 | 6.5 | 3 |
| Sph | 0.986 | 1 | 1.0 | 3 |
| dhSph | 0.270 | 1 | 2.1 | 3 |
| S1P | 0.243 | 1 | 0.8 | 3 |
| dhS1P | 0.835 | 1 | 0.0 | 3 |
| HexSph | 0.519 | 1 | 0.2 | 3 |
| LysoSM | 0.393 | 1 | 8.8 | 3 |
| C14 | 0.829 | 1 | 2.2 | 3 |
| C16 | 0.019 | 1 | 5.9 | 3 |
| C18 | 0.300 | 1 | 1.8 | 3 |
| C20 | 0.032 | 1 | 4.5 | 3 |
| C26 | 0.585 | 1 | 1.2 | 3 |
| C26:1 | 0.912 | 1 | 0.1 | 3 |
| HexC14 | 0.982 | 1 | 0.0 | 3 |
| HexC16 | 0.333 | 1 | 1.5 | 3 |
| HexC18 | 0.802 | 1 | 1.0 | 3 |
| HexC20 | 0.882 | 1 | 0.0 | 3 |
| HexC22 | 0.872 | 1 | 0.3 | 3 |
| HexC24 | 0.619 | 1 | 0.2 | 3 |
| HexC24:1 | 0.853 | 1 | 1.3 | 3 |
| HexC26 | 0.588 | 1 | 0.2 | 3 |
| HexC26:1 | 0.834 | 1 | 0.5 | 3 |
| SMC14 | 0.276 | 1 | 4.0 | 3 |
| SMC16 | 0.594 | 1 | 1.1 | 3 |
| SMC18 | 0.425 | 1 | 3.9 | 3 |
| SMC20 | 0.959 | 1 | 9.3 | 3 |
| SMC22 | 0.704 | 1 | 9.8 | 3 |
| SMC24 | 0.555 | 1 | 9.8 | 3 |
| SMC24:1 | 0.546 | 1 | 4.3 | 3 |
| SMC26 | 0.453 | 1 | 0.6 | 3 |
| SMC26:1 | 0.935 | 1 | 3.6 | 3 |
