# Supplementary material for: Large granular lymphocyte leukemia serum and corresponding hematological parameters reveal unique cytokine and sphingolipid biomarkers and associations with STAT3 mutations
Source: Cancer Med. 2020 Jul 25;9(18):6533–49. doi: 10.1002/cam4.3246 (PMC7520360; doi:10.1002/cam4.3246)
Supplement: Supplementary file 9 — Table S5 [file CAM4-9-6533-s009.docx]

**Supplementary Table 5. Regressions of cytokines or sphingolipids against MCV and STAT3 mutation.** Slopes and p-values from regression models of log-transformed cytokine or sphingolipid data against MCV with an interaction with STAT3 mutation status are reported. No p-values were significant, therefore no multiple testing corrections were applied.

| **Cytokine or lipid** | **Slope WT STAT** | **Slope Mut STAT** | **p-value** |
| --- | --- | --- | --- |
| IL-1RA | -0.022 | 0.037 | 0.054 |
| IL-10 | -0.015 | 0.028 | 0.058 |
| HexC16 | 0.010 | -0.006 | 0.092 |
| S1P | 0.011 | 0.001 | 0.105 |
| TGFB2 | -0.002 | 0.011 | 0.113 |
| HexC24:1 | 0.010 | -0.005 | 0.160 |
| HexC20 | 0.005 | -0.006 | 0.201 |
| HexC26:1 | 0.001 | -0.024 | 0.210 |
| SMC26:1 | 0.010 | -0.002 | 0.211 |
| SMC24 | 0.008 | -0.003 | 0.213 |
| Sph | 0.012 | -0.003 | 0.217 |
| TGFB1 | 0.001 | 0.012 | 0.225 |
| HexC22 | 0.004 | -0.007 | 0.235 |
| Flt-3 Ligand | -0.030 | 0.010 | 0.238 |
| SMC16 | 0.015 | 0.006 | 0.244 |
| LysoSM | 0.013 | 0.003 | 0.266 |
| TRAIL | 0.022 | -0.001 | 0.266 |
| sFas-Ligand | 0.018 | 0.000 | 0.273 |
| sFas | -0.004 | 0.006 | 0.282 |
| HexC24 | 0.002 | -0.010 | 0.292 |
| SMC24:1 | 0.015 | 0.005 | 0.311 |
| Eotaxin-2 | 0.000 | 0.009 | 0.341 |
| HexC26 | 0.003 | -0.016 | 0.341 |
| dhS1P | 0.011 | 0.003 | 0.350 |
| HexC14 | 0.003 | -0.006 | 0.362 |
| HexC18 | -0.002 | -0.009 | 0.368 |
| SDF-1a+b | 0.008 | 0.015 | 0.370 |
| SMC26 | 0.008 | 0.000 | 0.403 |
| IL-6 | -0.009 | 0.018 | 0.422 |
| SMC22 | 0.009 | 0.001 | 0.426 |
| dhSph | 0.009 | -0.002 | 0.442 |
| IP-10 | -0.008 | 0.006 | 0.463 |
| C26 | -0.003 | 0.003 | 0.478 |
| sVCAM-1 | 0.013 | 0.004 | 0.499 |
| C24 | 0.002 | -0.005 | 0.508 |
| C18 | -0.005 | 0.001 | 0.576 |
| IL-8 | -0.002 | 0.016 | 0.609 |
| IL-18 | 0.012 | 0.002 | 0.616 |
| C16 | 0.002 | -0.002 | 0.643 |
| IFNa2 | 0.001 | 0.012 | 0.644 |
| SMC20 | 0.007 | 0.003 | 0.645 |
| IFNg | 0.020 | 0.007 | 0.683 |
| C20 | -0.003 | 0.001 | 0.710 |
| MIG | 0.016 | 0.026 | 0.731 |
| EGF | 0.018 | 0.022 | 0.759 |
| RANTES | 0.014 | 0.012 | 0.811 |
| G-CSF | -0.002 | 0.005 | 0.820 |
| MIP-1b | 0.008 | 0.013 | 0.846 |
| C22 | -0.001 | -0.003 | 0.854 |
| C14 | -0.003 | -0.004 | 0.859 |
| SMC14 | 0.004 | 0.003 | 0.873 |
| HexSph | 0.015 | 0.013 | 0.899 |
| C26:1 | 0.006 | 0.005 | 0.914 |
| sICAM-1 | 0.001 | 0.000 | 0.918 |
| C24:1 | 0.003 | 0.004 | 0.938 |
| MIP-3b | -0.005 | -0.004 | 0.954 |
| SMC18 | 0.007 | 0.006 | 0.958 |
